# A missense mutation in *Ehd1* associated with defective spermatogenesis and male infertility

**DOI:** 10.3389/fcell.2023.1240558

**Published:** 2023-10-12

**Authors:** Katrin Meindl, Naomi Issler, Sara Afonso, Alberto Cebrian-Serrano, Karin Müller, Christina Sterner, Helga Othmen, Ines Tegtmeier, Ralph Witzgall, Enriko Klootwijk, Benjamin Davies, Robert Kleta, Richard Warth

**Affiliations:** ^1^ Medical Cell Biology, University Regensburg, Regensburg, Germany; ^2^ Department of Renal Medicine, University College London, London, United Kingdom; ^3^ Pediatric Nephrology Unit and Research Lab, Hadassah Medical Center and Faculty of Medicine, Hebrew University of Jerusalem, Jerusalem, Israel; ^4^ Institute of Cellular and Molecular Physiology, Friedrich-Alexander-Universität Erlangen-Nürnberg, Erlangen, Germany; ^5^ Wellcome Centre for Human Genetics, University Oxford, Oxford, United Kingdom; ^6^ Helmholtz Zentrum München, Institute of Diabetes and Obesity, Munich, Germany; ^7^ German Center for Diabetes Research (DZD), Neuherberg, Germany; ^8^ Leibniz Institute for Zoo- und Wildlife Research, Berlin, Germany; ^9^ Molecular and Cellular Anatomy, University Regensburg, Regensburg, Germany; ^10^ Genetic Modification Service, The Francis Crick Institute, London, United Kingdom

**Keywords:** testis, endocytosis, retromer, genetic disease, sperm, ciliogenesis

## Abstract

Normal function of the C-terminal Eps15 homology domain-containing protein 1 (EHD1) has previously been associated with endocytic vesicle trafficking, shaping of intracellular membranes, and ciliogenesis. We recently identified an autosomal recessive missense mutation c.1192C>T (p.R398W) of EHD1 in patients who had low molecular weight proteinuria (0.7–2.1 g/d) and high-frequency hearing loss. It was already known from *Ehd1* knockout mice that inactivation of *Ehd1* can lead to male infertility. However, the exact role of the EHD1 protein and its p.R398W mutant during spermatogenesis remained still unclear. Here, we report the testicular phenotype of a knockin mouse model carrying the p.R398W mutation in the EHD1 protein. Male homozygous knockin mice were infertile, whereas the mutation had no effect on female fertility. Testes and epididymes were significantly reduced in size and weight. The testicular epithelium appeared profoundly damaged and had a disorganized architecture. The composition of developing cell types was altered. Malformed acrosomes covered underdeveloped and misshaped sperm heads. In the sperm tail, midpieces were largely missing indicating disturbed assembly of the sperm tail. Defective structures, i.e., nuclei, acrosomes, and sperm tail midpieces, were observed in large vacuoles scattered throughout the epithelium. Interestingly, cilia formation itself did not appear to be affected, as the axoneme and other parts of the sperm tails except the midpieces appeared to be intact. In wildtype mice, EHD1 co-localized with acrosomal granules on round spermatids, suggesting a role of the EHD1 protein during acrosomal development. Wildtype EHD1 also co-localized with the VPS35 component of the retromer complex, whereas the p.R398W mutant did not. The testicular pathologies appeared very early during the first spermatogenic wave in young mice (starting at 14 dpp) and tubular destruction worsened with age. Taken together, EHD1 plays an important and probably multifaceted role in spermatogenesis in mice. Therefore, *EHD1* may also be a hitherto underestimated infertility gene in humans.

## Introduction

### Infertility

Infertility is a major health problem worldwide and its incidence is increasing (Rutstein and Shah, 2004; Pereira and Sousa, 2023; WHO, 2023). It is defined as a failure to achieve pregnancy after 12 months or more of regular unprotected sexual intercourse (Zegers-Hochschild et al., 2009). 20%–50% of infertility cases can been attributed to male factors (Kumar and Singh, 2015) and several causes are known to contribute to male infertility. These include hormonal imbalances, structural changes in the reproductive organs, exposure to toxic substances, sexually transmitted infections, and genetic factors (Babakhanzadeh et al., 2020). The latter are thought to be responsible for 15%–30% of cases of male infertility (Shamsi et al., 2011; Krausz and Riera-Escamilla, 2018).

### Stages of spermatogenesis

**FIGURE 1:**
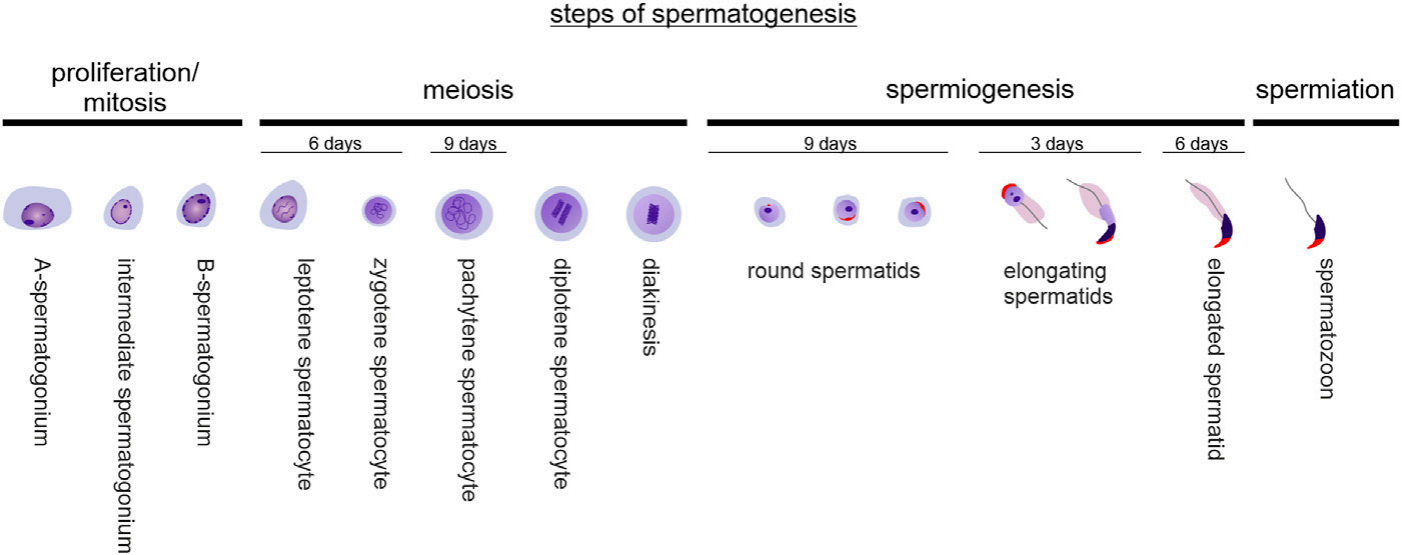
Steps of spermatogenesis. Spermatogenesis is composed of spermatogonial mitotic divisions for expansion, two successive meiotic reductive divisions, complex differentiation processes of round spermatids in order to become elongated spermatids (spermiogenesis), and spermiation - the detachment of mature spermatozoa from the seminiferous epithelium. In mice, it takes approx. 35 days for a spermatozoon to originate from a spermatogonium.

Spermatogenesis is a highly complex, multistep and tightly orchestrated process of sperm production that takes place in the seminiferous tubules of the testis (Figure 1). Spermatogonia continuously proliferate by mitotic division before becoming spermatocytes, which then undergo two more rapid meiotic divisions and result in haploid round spermatids. From this point on, no further division occurs: Instead, immature spermatids undergo complex cyto-differentiation (spermiogenesis) to become streamlined, functional, and motile spermatozoa that are released into the tubular lumen in a process called spermiation (O'Donnell, 2014). In mice, based on the composition of the different cell types found in the epithelium of the seminiferous tubules at the same time, 12 distinct tubular stages can be distinguished (Oakberg, 1956). Along the length of a seminiferous tubule, different stages are present (Supplementary Figure S1). The duration of one spermatogenic cycle (period until the same tubular stage appears again at the same place) is approximately 8.6 days in mice, and approximately 35 days are required for a spermatogonium to develop into a haploid spermatozoon (Oakberg, 1956; Griswold, 2016). In humans, the duration of one spermatogenic cycle is 16 days and the time required for a spermatogonium to develop into a haploid spermatozoon ∼68 days, divided into six stages (Amann, 2008; Huhtaniemi and Winters, 2017).

### Endocytic recycling protein EHD1

Endocytosis and intracellular vesicle dynamics are essential for cell life and survival: to receive nutrients from outside, to transport membranes and vesicle content between different compartments, to grow and polarize, to internalize surface receptors, and to maintain homeostasis and cellular integrity (Conner and Schmid, 2003; Di Fiore and von Zastrow, 2014). Dynamin-like C-terminal Eps15 homology domain (EHD)-containing protein 1 (EHD1) is one of four known mammalian EHD proteins (Mintz et al., 1999). They are all thought to regulate various steps of endocytic transport, such as endosomal cleavage upon ATP hydrolysis (Daumke et al., 2007), vesicle transport, and receptor recycling, as they are localized to cytoplasmic vesicles in close proximity to endosomes and the Golgi apparatus (Mintz et al., 1999). These functions are mainly accomplished through cooperation with various binding partners (Zhang et al., 2012b), including members of the Rab family of small GTPases, which are known to be important regulators of endocytic trafficking (Naslavsky and Caplan, 2011). EHD1 is widely expressed in various human tissues, with highest expression in the brain, respiratory system, kidney, and male reproductive system, particularly the testes (https://www.proteinatlas.org/ENSG00000110047-EHD1/tissue) (Mintz et al., 1999). In addition to its important role in endocytosis, there is strong evidence that EHD1 is involved in other membrane-shaping processes (Naslavsky and Caplan, 2022) and in ciliogenesis (Lu et al., 2015).

Endocytosed material is passed through different parts of the endosomal system, e.g., early endosomes, recycling endosomes, late endosomes, and/or lysosomes, before finally reaching the intended destination (McKenzie et al., 2012). The trans-Golgi network (TGN) connects the Golgi apparatus to the endosomal system and accomplishes bidirectional transport processes that form the basis for the intensive exchange between these two compartments (Progida and Bakke, 2016). In this context, the retromer complex has gained increasing attention in the recent years (Seaman et al., 1998). In mammals, the retromer complex is important for the regulation of retrograde transport, which describes the transport of cargos (proteins, lipids, toxins) from the plasma membrane to the Golgi via the endosomal system and the trans-Golgi network as well as transport from early endosomes to the lysosomal pathway (McKenzie et al., 2012). The retromer is a multiprotein complex which is built up of two main subcomplexes in mammals: Subcomplex one consists of three VPS orthologs (VPS35, VPS26, and VPS29) that bind cargo, while subcomplex two includes the sorting enzymes 1 and 2 (SNX1 & 2) and is involved in membrane tubulation (Gokool et al., 2007). EHD1 was reported to interact with VPS26, and the absence of EHD1 resulted in destabilization of SNX1-positive intracellular tubular membrane structures. It was therefore concluded that EHD1 facilitates the retrieval of endosomal vesicles to the Golgi (Gokool et al., 2007). How might this be relevant to spermiogenesis? It is known that acrosome biogenesis and growth depend on the delivery of proteins and membranes from the Golgi (Da Ros et al., 2015) and involve components of the endosomal system (Berruti and Paiardi, 2011). It is conceivable that EDH1 plays a role at the junction of the endosomal system and the Golgi and thus in acrosome biogenesis and growth.

### Mutated EHD1 impairs spermatogenesis and fertility

During spermiogenesis, profound remodeling of maturing cells occurs: Round spermatids begin to build important structures such as the acrosome and sperm tail, while changing their nuclear and cytoplasmic shape to become elongated spermatozoa that are motile and able to fertilize an egg. Disruptions in any of these processes can lead to impaired or complete loss of fertility (O'Donnell, 2014). Many processes during spermatogenesis are dependent on endocytic activity, including the development of the acrosome, a lysosome-derived structure that covers nearly 2/3 of the sperm head and allows penetration into the zona pellucida of the oocyte. In addition, sperm tails represent a special class of motile cilia showing the typical axonemal “9x2 + 2” structure. Cilia development is based on endocytic vesicle transport and membrane remodeling and EHD1 is thought to play an important role in this process (Lu et al., 2015). The integrity of the so-called blood-testis barrier is important in protecting maturing germ cells in the seminiferous epithelium. This barrier, established by neighboring Sertoli cells, divides the seminiferous epithelium into a basal and an adluminal compartment (Cheng and Mruk, 2012). The blood-testis barrier is not a static structure, as it undergoes massive remodeling during spermatogenesis to allow developing germ cells to pass. The opening and closing of the blood-testis barrier depends on endocytosis, endosome-mediated degradation, transcytosis and recycling (Cheng and Mruk, 2012) and thus may involve EHD1-dependent mechanisms.

Recently, we identified a homozygous missense mutation in the gene encoding the EHD1 protein in patients with low-molecular-weight proteinuria (0.7–2.1 g/d) and high-frequency hearing loss (Issler et al., 2022). To gain further insights into the pathophysiology of the mutant EHD1 protein, we established a knockin mouse model carrying the respective mutation (p.R398W) in the mouse *Ehd1* gene. Breeding of these *Ehd1* ^*R398W/R398W*^ mice revealed that the male homozygous mice were unable to produce offspring (Issler et al., 2022), but the testis phenotype has not been previously investigated. Interestingly, male infertility was also observed in *Ehd1* knockout mice. Male *Ehd1* knockout mice survived at a sub-Mendelian rate, were infertile, had smaller testes and massive defects at multiple steps of spermatogenesis. The normal spermatogenesis of *Ehd1* knockout mice was disrupted, acrosome development in round spermatids was abnormal and normal elongated spermatids were absent (Rainey et al., 2010).

Here, we examined the effects of the homozygous p.R398W missense variant of EHD1 found in patients with proteinuria and hearing loss on spermatogenesis in mice. The testicular tissue of *Ehd1* ^*R398W/R398W*^ mice was severely damaged. Spermatogenesis was disrupted at multiple levels and led to infertility of male mice. Since spermatogenesis in mice and humans shows great similarities, it is reasonable to assume that mutations in *EHD1* can also lead to failure of spermatogenesis and infertility in humans.

## Methods

### Ehd1^R398W/R398W^ knockin mice

*Ehd1* ^*R398W/R398W*^ knockin mice were generated by the Wellcome Centre for Human Genetics, University of Oxford, United Kingdom using Crispr/Cas technology. The breeding of the hybrid mice was carried out in accordance with United Kingdom Home Office Animal (Scientific Procedures) Act 1986, with procedures reviewed by the Clinical Medicine Animal Welfare and Ethical Review Body at the University of Oxford, and conducted under project license PPL 30/3437 and 30/3085. For detailed information see recently published data (Issler et al., 2022). Experiments were performed according to the guidelines for the care and use of laboratory animals published by the US National Institutes of Health. Animal experiments on mice were approved by the “Regierung Unterfranken”, Germany. Animals backcrossed into the SV129 genetic background for more than five generations were provided with food and water *ad libitum* and maintained on a 12 h light:12 h dark cycle. Experimental groups were determined by genotype and were therefore not randomized, with no animals excluded from the analysis. Sample sizes were selected on the basis of previously published studies (Davies et al., 2016; Issler et al., 2022), and all phenotypic characterizations were performed blind to experimental group where appropriate. Before tissue removal, mice were sacrificed by decapitation under deep anaesthesia (2.5% isoflurane).

### Sex determination of newborn mice by PCR

Reliable sex determination of newborn mice (1 day *postpartum*) was performed by polymerase chain reaction (PCR) using a primer pair that amplifies fragments of the X and Y chromosomes with a significant size difference between the respective amplicons, as described by (McFarlane et al., 2013). gDNA was amplified using the following primer pairs: SX_F: GAT​GAT​TTG​AGT​GGA​AAT​GTG​AGG​TA, SX_R: CTT​ATG​TTT​ATA​GGC​ATG​CAC​CAT​GTA, Zfy_F: GAC​TAG​ACA​TGT​CTT​AAC​ATC​TGT​CC, Zfy_R: CCT​ATT​GCA​TGG​ACT​GCA​GCT​TAT​G. PCR reactions were performed using the following protocol: First denaturation step at 94°C for 3°min, 35 cycles of 94°C for 30°s, 55°C for 30°s and 72°C for 30°s, followed by a final elongation at 72°C for 4 min. PCR products were separated by gel electrophoresis using a 2% agarose gel.

### Tissue fixation, paraffin embedment and sectioning

To achieve optimal preservation of testicular and epididymal tissues, organs were harvested and post-fixed in Bouin’s solution (10% formalin, aqueous picric acid 0.95%, glacial acetic acid 4.7%) for 24 h at 4°C. This fixation method preserved testicular and epididymal tissue integrity and allowed identification of mitotic and meiotic figures. Then, the tissue was dehydrated stepwise for 25 min each in 70%/80%/90%/100% methanol and then in 100% propan-2-ol for 25 min. The tissue was then incubated in a 1:1 mixture of propan-2-ol and liquid paraffin (64°C, 1 h) and stored overnight in pure paraffin (64°C). The next day, the tissue was incubated in fresh paraffin for an additional 60 min before blocking out. 5-µm sections were mounted on Poly-Lys slides (for histological and immunofluorescence staining; O. Kindler GmbH) or Superfrost^®^ slides (for RNAScope experiments; Thermo Scientific) and air-dried at 40°C for 30 min. The paraffin sections were stored at 4°C until use.

### Isolation of mature sperms from cauda epididymis

Testis and epididymis were carefully removed and separated from each other. The caudal part of the epididymis was separated from the caput and corpus, incised several times with small scissors, and spermatozoa were washed out in 500 µL GM105 SpermAir (Gynemed, Lensahn, Germany), a ready-to-use medium for all sperm preparations, sperm washes, and swim-up tests. After 5 min of incubation, 100 µL of the supernatant was transferred to a new cup containing 500 µL of fresh GM105 SpermAir medium.

### Immunofluorescence staining of isolated sperms from cauda epididymis

Isolated spermatozoa stored in GM105 SpermAir medium were centrifuged (60 g) for 10 min and the pellet was carefully resuspended in a fixation solution containing 3% paraformaldehyde, 100 mM sucrose, 90 mM NaCl, 15 mM K_2_HPO_4_, 1 mM EGTA, and 2 mM MgCl_2_ (pH 7.4) and incubated for 5 min. After removal of the fixative solution, spermatozoa were washed three times with PBS. Epitopes were unmasked with 0.1% SDS in PBS for 5 min. Primary antibodies were diluted in a PBS-based antibody dilution solution (0.5% BSA, 0.04% TritonX-100), and sperm were incubated in the primary antibody containing solution for 1 h at room temperature. Then, spermatozoa were washed three times with PBS, centrifuged (60 g, 5 min), and incubated for another 60 min in the dark in secondary antibody dilution (0.5% BSA, 0.04% TritonX-100). Finally, the stained and washed spermatozoa were pelleted by centrifugation, resuspended in a very small amount of PBS and covered with lamellae on a glass slide.

### Hematoxylin/Eosin (H.E.) staining

H.E. staining was performed on 5 µm thick paraffin sections. After deparaffinization and rehydration twice in xylene, 99% isopropanol, and decreasing concentrations of ethanol (95%, 80%, 70%) for 12 min each, sections were washed in PBS for 5 min and incubated in Mayer’s Hemalum for 3 min. Excess staining solution was removed by washing with running tap water until the water remained clear. For counterstaining, the sections were incubated in 2% alcoholic eosin solution for 2 min, followed by short dehydration steps in ethanol of increasing concentrations (70%, 80%, 90%), 99% isopropanol, and a final incubation in xylene (2 × 5 min). The slides were mounted in DePeX mounting medium (Serva Electrophoresis GmbH, Heidelberg, Germany). Analysis was performed using an inverted microscope (Observer.Z1, Zeiss).

### Immunofluorescence

Immunofluorescence stains were performed on 5 µm paraffin sections. After deparaffinization and rehydration twice in xylene, 99% isopropanol, and decreasing concentrations of ethanol (95%, 80%, 70%) for 12 min each, sections were washed in PBS for 5 min, and epitopes were unmasked by boiling in citrate buffer (pH 6.0) at 95°C for 15 min. Nonspecific antibody binding sites were saturated with PBS-based blocking solution (5% bovine serum albumin (BSA), 0.04% TritonX-100). Primary antibodies were diluted in PBS-based antibody dilution solution (0.5% BSA, 0.04% TritonX-100), and tissue sections were coated with the appropriately diluted primary antibodies and incubated overnight in a humidity chamber at 4°C. On the next day, sections were washed in PBS for 5 min and incubated with diluted secondary antibodies for 1 h in the dark at room temperature. Finally, the stained sections were mounted with non-fluorescent glycerol gel mounting medium (DAKO). The following antibodies and fluorescent dyes were used for immunofluorescence stains: anti-EHD1, rabbit monoclonal [EPR4954] IgG (1:100) (Catalog number: ab109311) (Abcam, Cambridge, United Kingdom); anti-SUN5 (SPAG4L), rabbit polyclonal IgG (1:50; Cat Nr. 17495-1-AP) (Proteintech, Planegg-Martinsried, Germany); anti-VPS35, goat polyclonal IgG (1:200 (Catalog number: ab10099); Abcam, Cambridge, United Kingdom); LectinPNA Alexa Fluor 647 conj. (1:650) (Catalog number: L32460) (Molecular Probes, Eugene, Oregon, US); HOE33342 dilution (Invitrogen, Karlsruhe, Germany). As secondary antibodies, AlexaFluor^®^ conjugated antibodies were used at 1:400 dilution (Thermo Fisher Scientific, Dreieich, Germany). The slides were mounted in DAKO Glycergel mounting medium (Agilent Technologies Deutschland GmbH, Waldbronn, Germany). Analysis was performed using an inverted microscope (Observer.Z1, Zeiss) or confocal microscopes (LSM710 and LSM980 with AiryScan, Zeiss).

#### Tissue preparation for electron microscopy

Testes and epididymides were transferred to a fixative solution containing 2% glutaraldehyde in 0.1 M Na-cacodylate (pH 7.4) and stored until further processing. Tissues were post-fixed by rinsing with 0.1 M Na-cacodylate pH 7.4 (3 × 20 min), 1% OsO_4_ in 0.1 M Na-cacodylate pH 7.4 (2 h), 0.1 M Na-cacodylate pH 7.4 (3 × 20 min) and dehydrated in increasing concentrations of ethanol 50%/70%/90%/96%/100% (20 min each) and followed by acetone (3 × 15 min). Finally, samples were embedded in Epon and polymerized at 60°C for 48 h. Ultrathin sections were examined using an EM902 transmission electron microscope at 80 keV (Zeiss, Oberkochen, Germany). Digital micrographs were acquired using a CCD-2k camera (Troendle, Moorenweis, Germany).

### *RNAScope®* experiments

*RNAScope®* experiments were performed on Bouin-fixed, paraffin-embedded testicular tissue using the *RNAScope® 2.5 HD Assay* Kit (ACD) according to the manufacturer’s instructions.

### Inducible LLC-PK1 cell model

A porcine proximal tubular kidney cell line (LLC-PK1) was used as the main cell culture model to investigate various aspects of EHD1 interactions and function. LLC-PK1 cells were stably transfected with either human wildtype EHD1 or mutant EHD1^R398W^ as published elsewhere (Issler et al., 2022). In brief, gene expression was controlled by a Tet-On system, i.e., addition of tetracycline (1 μg/mL) to the medium for 48 h results in activated expression of either wildtype EHD1 or EHD1^R398W^. Cells were cultured in small flasks at 37°C and 5% CO_2_ in appropriate LLC-PK1 medium and split when confluent. For immunofluorescence staining, 300.000 cells per well were seeded on glass coverslips in 6-well plates and EHD1 expression was induced for 48 h with tetracycline.

### Immunofluorescence stains of LLC-PK1 cells

After tetracycline induction for 48 h, the medium was removed, and the cells were washed three times with PBS and fixed with a fixation solution containing 3% paraformaldehyde, 100 mM sucrose, 90 mM NaCl, 15 mM K_2_HPO_4_, 1 mM EGTA, and 2 mM MgCl_2_ (pH 7.4) for 5 min. For epitope unmasking, fixed cells were incubated with 0.1% SDS in PBS for 5 min at room temperature. Nonspecific antibody binding sites were saturated with PBS-based blocking solution (5% BSA, 0.04% TritonX-100). Cells were incubated sequentially in the primary and secondary antibody dilutions for 1 h at room temperature and washed in PBS after each incubation. Antibodies were diluted in a PBS-based antibody dilution solution (0.5% BSA, 0.04% TritonX-100). After final washing, glass coverslips were mounted on slides with non-fluorescent glycerol gel mounting medium (DAKO). Cells were analyzed using an inverted microscope (Observer.Z1, Zeiss).

## Statistics

Data are shown as mean values ±standard error of the mean (SEM); “n” stands for the number of observations. Two-way ANOVA with Tukey’s multiple comparison test (GraphPad Prism 9.5.1) was used to calculate significance between different groups. A *p*-value ≤0.05 was accepted to indicate statistical significance.

## Results

### Appearance of male reproductive organs in *Ehd1* ^*R398W/R398W*^ mice

Homozygous *Ehd1* ^*R398W/R398W*^ male mice were infertile, failing to produce offspring when mated with heterozygous *Ehd1* ^*R398W/wt*^ females or wildtype females. However, female homozygous *Ehd1* ^*R398W/R398W*^ mice were fertile, indicating that the pathological effects of the mutation were restricted to the male reproductive tract. Recently, detailed breeding statistics have been published (Issler et al., 2022).

**FIGURE 2:**
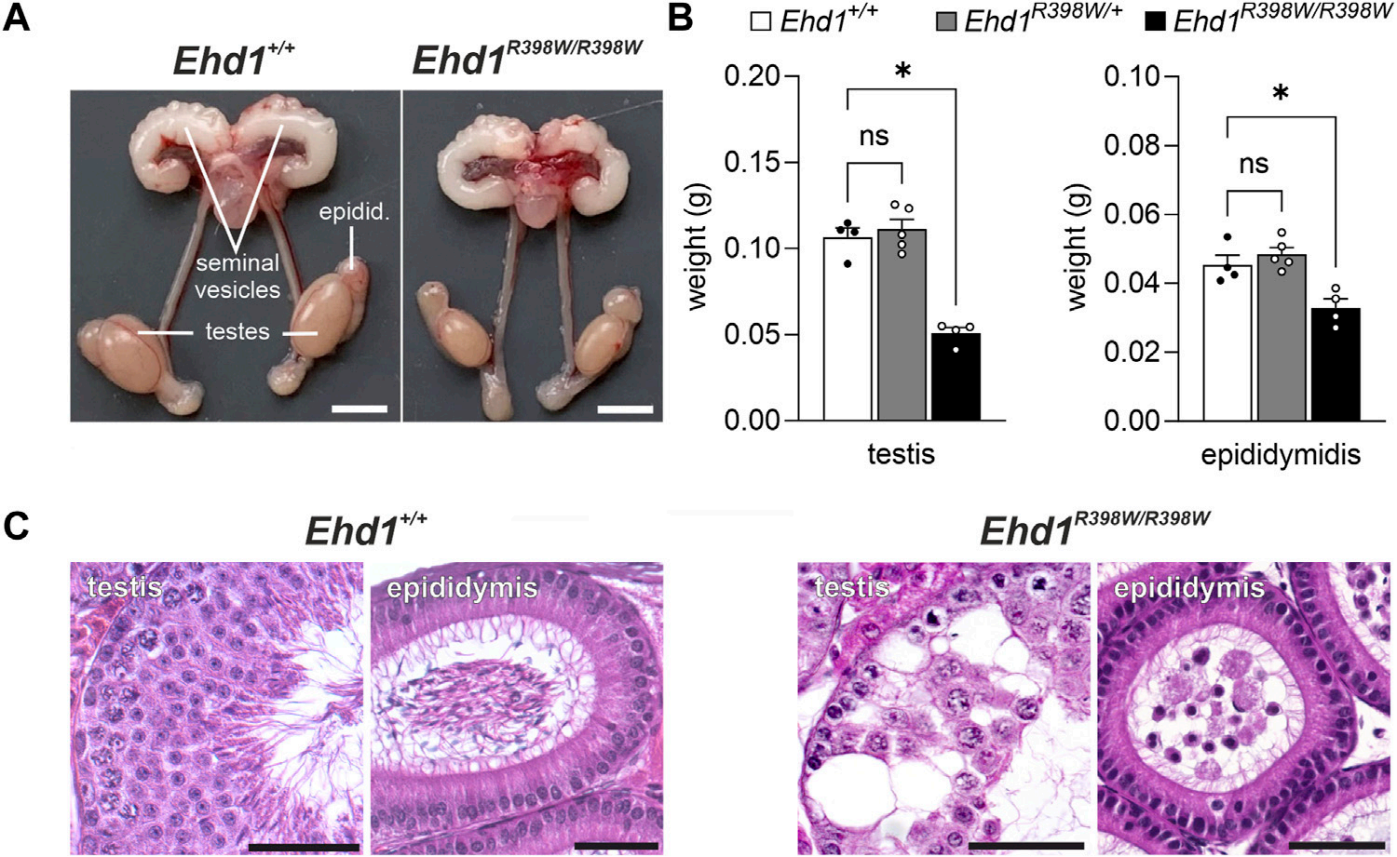
Testicular phenotype of *Ehd1* ^*R398W/R398W*^ mice. **(A)** Male *Ehd1* ^R398W/R398W^ mice had testes of smaller size. Scale bars: 0.5 cm. **(B)** Summary of testis and epididymis weights (mean values of the left and right organs for each animal). Asterisks indicated *p* < 0.05. Tissue weights of heterozygote *Ehd1* ^R398W/+^ mice were not different (ns) from those of wildtype mice. Error bars represent SEM. **(C)** Massive destruction of the seminiferous epithelium associated with the presence of immature sperm precursors in the epididymis of *Ehd1* knockin mice. Scale bars: 50 µm.

Macroscopically, the male reproductive organs showed a marked difference in the size and weight of the testes of *Ehd1* ^*R398W/R398W*^ mice compared to wildtype animals. Interestingly, the seminal vesicles did not appear to be affected (Figures 2A,B). To gain insights on the microscopic level, we performed hematoxylin/eosin (H.E.) staining on Bouin-fixed paraffin-embedded testis and epididymis tissues (Figure 2C). A Periodic acid-Schiff’s stain is shown in Supplementary Figure S2. We found severe destruction in almost all cross-sections of seminiferous tubules from *Ehd1* ^*R398W/R398W*^ mice, which exhibited large vacuoles, as well as an abnormal distribution and reduced number of cells. Instead of typical streamlined spermatozoa, a large number of immature cells were observed in the lumina of the epididymal ducts, indicating premature detachment from the testicular epithelium. Because of this severe pathology, no clear stage definition of testicular tubules was possible. Heterozygous mice, however, showed a normal testicular histology (Supplementary Figure S3).

### EHD1 expression in the seminiferous epithelium during spermatogenesis is stage-dependent

**FIGURE 3:**
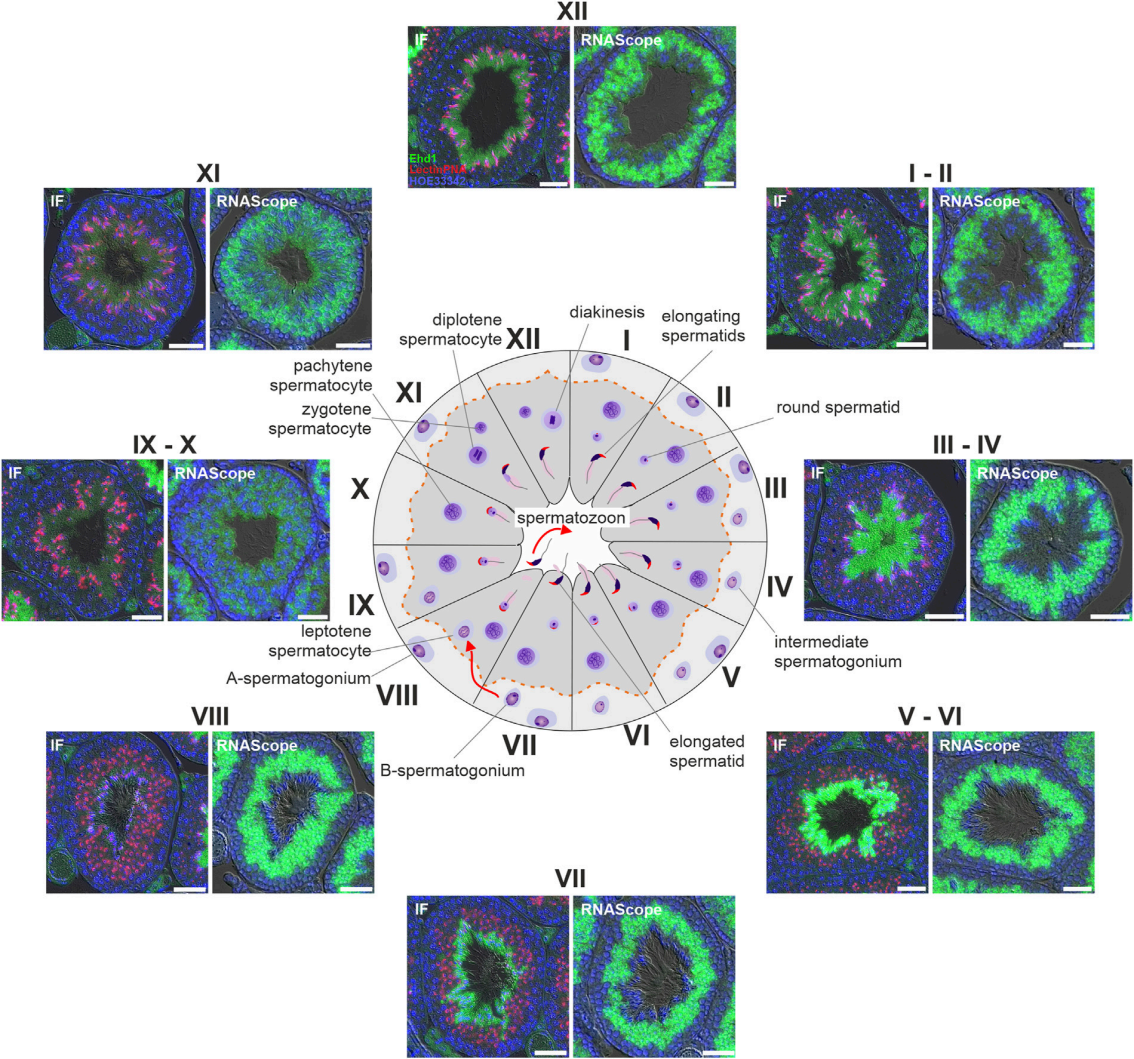
EHD1 expression during spermatogenesis is stage-dependent. Immunofluorescence (IF) and RNAScope^®^ was performed on Bouin-fixed, paraffin-embedded tissue. Depicted are consecutive sections of the same tubules for different stages of spermatogenesis (marked by roman numerals). EHD1 protein (green, IF pictures) showed a stage-dependent expression pattern during the seminiferous cycle with peak intensity in the cytoplasm of matured, elongated spermatids near the lumen and right before spermiation taking place in stage VII-VIII, while EHD1 mRNA (green, RNAScope pictures) was mainly located in near proximity to round spermatids in the middle of the epithelium. The discrepancy between mRNA and protein expression is probably due to so-called translational delay. Spermatogenic stages were assessed using LectinPNA staining of the acrosomes (red, IF pictures) referring to the scheme of Meistrich and Hess (Meistrich and Hess, 2013). The dotted red line in the center scheme indicates the blood-testis barrier. Green: EHD1 protein or mRNA, red: LectinPNA (acrosome), blue: HOE33342 (nuclei). Scale Bar: 50 µm.

The expression of EHD1 is particularly high in testicular tissue (Mintz et al., 1999). To obtain clues about the functional importance of EHD1, we first investigated in which cells and at which time point of spermatogenesis EHD1 mRNA and protein are expressed. We used RNAScope technology and immunofluorescence antibody staining on consecutive sections of Bouin-fixed paraffin-embedded testicular tissue. This approach allowed us to compare mRNA expression and protein expression on identical tubules (Figure 3). Overview images of two consecutive sections are shown in Supplementary Figure S4. Based on the individual composition of the different cell types combined with the observed acrosomal shape visualized by LectinPNA staining, the stages of the tubule cross sections were identified using the guidelines of Meistrich and Hess (Meistrich and Hess, 2013) (see also Supplementary Figure S1). The EHD1 protein showed a stage-dependent expression pattern within the sperm epithelium and was mainly present in the adluminal compartment. During stages III-VI, expression reached its maximum and was concentrated in the mature spermatids toward the lumen (Figure 3). After successful detachment of spermatids from the epithelium (stage VIII), little protein was detectable until stage XI. EHD1 mRNA expression was fundamentally different from protein expression and virtually no overlap was observed. The mRNA of EHD1 was mainly concentrated in the region of round spermatids localized in the middle of the epithelium and appeared to be expressed much more consistently than protein throughout the spermatogenesis cycle. Only at stages IX-X reduced mRNA expression was detected.

**FIGURE 4:**
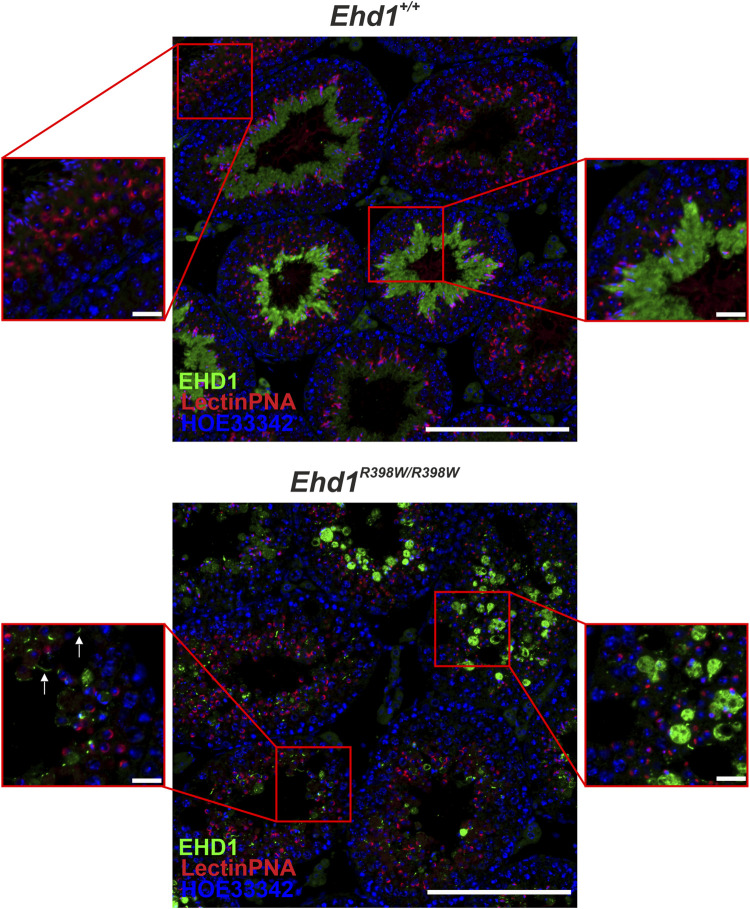
EHD1 expression in the testes of *Ehd1* ^*+/+*^ and *Ehd1* ^*R398W/R398W*^ mice. In *Ehd1* ^+/+^ mice, EHD1 (green) showed the typical stage-dependent expression pattern. While it was completely absent immediately after spermiation (left zoom picture, upper line), its expression increased with ongoing spermatogenesis (right zoom picture, upper line) and showed peak expression in stage VI-VII (not shown). In *Ehd1* ^R398W/R398W^ mice, the mutant protein was either observed as it built thin tubules (arrows in the lower left magnified image section), or formed bubble-shaped structures (lower right magnified image section). Green: EHD1; red: LectinPNA (acrosomes); blue: HOE33342 (nuclei). Scale bar: 200 µm (overview images), 20 µm (magnified images).

Direct comparison of two overview images of *Ehd1* ^*+/+*^ and *Ehd1* ^*R398W/R398W*^ mice (Figure 4) revealed the altered expression and localization of the mutant EHD1 protein. In wild-type animals, the expression level and localization was dependent on the stages of spermatogenesis. In the *Ehd1* ^*R398W/R398W*^ mice, this assignment was not possible because the stages could no longer be clearly identified due to the pathological changes. The mutant EHD1 protein was found in large, round, vesicular structures - presumably cytoplasmic vesicles/autolysosomes, some of which harbored nuclei and defective acrosomal structures. In addition, the mutant EHD1 protein was located in long tubular structures that could not be assigned to any particular cell type.

**FIGURE 5:**
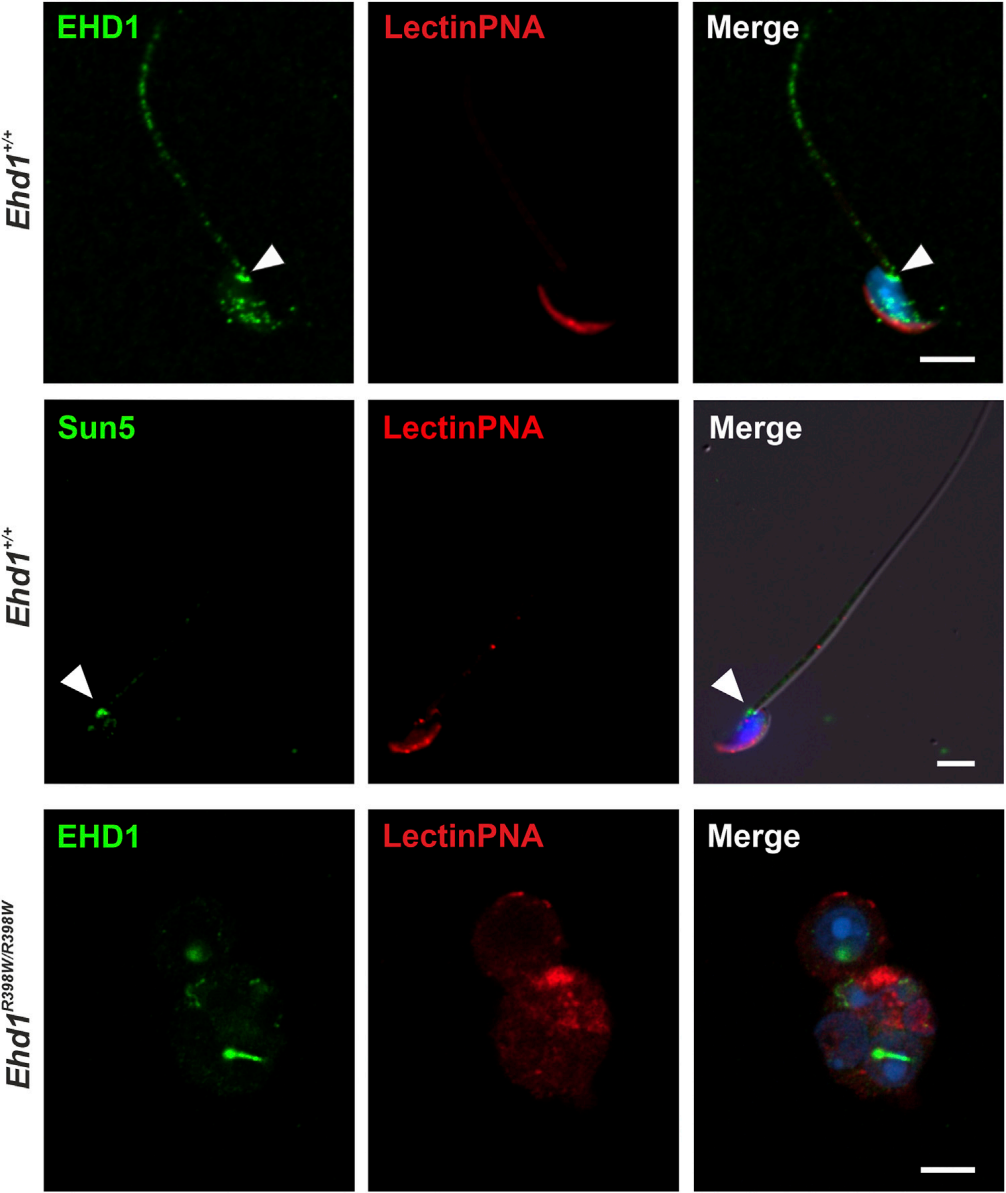
EHD1 is present in epididymal sperm. Confocal images revealed EHD1 (green) localization at the acrosome (red), the head-to-tail coupling apparatus (HTCA, arrowhead), and the sperm tail midpiece in *Ehd1* ^+/+^ mice (top panel). HTCA was also labeled with an anti-SUN5 antibody (SUN5 is a marker protein of HTCA; middle panel) (Shang et al., 2017). In the epididymides of knockin mice, normal sperm were virtually absent (lower panel). Instead, abnormal cells were found. Green: EHD1, red: LectinPNA (acrosomes), blue: HOE33342 (nuclei). Scale bar: 5 µm.

Having studied the localization of the protein in the seminiferous epithelium, we investigated if EHD1 protein is present in spermatozoa that had already been transported to the caudal part of the epididymis (Figure 5). Wildtype spermatozoa showed a clear co-localization of EHD1-positive spots at the acrosome, at the neck/head-to-tail coupling apparatus (HTCA) (Shang et al., 2017; Tapia Contreras and Hoyer-Fender, 2019; Wu et al., 2020), and in the middle region of the sperm tail, the part of the tail responsible for supplying energy to drive the flagellar beat. Whether EHD1 in epididymal sperm is just a remnant from the maturation process or whether it still serves an important function remains to be investigated.

Normal spermatozoa were virtually absent in the epididymis of *Ehd1* ^*R398W/R398W*^ mice. Instead, we found undifferentiated and defective round spermatids that prematurely detached from the seminiferous epithelium and were transported to the epididymis. The acrosome-derived remnants agglomerated in the region close to the nucleus, although no clear assignment to a specific phase of acrosomal development was possible. These results suggest that dysfunction of EHD1 leads to malformation of the acrosome and that normal EHD1 function is likely essential for the development of a fully functional acrosome.

### The p.R398W mutation of EHD1 affects already the early postnatal testicular development

**FIGURE 6:**
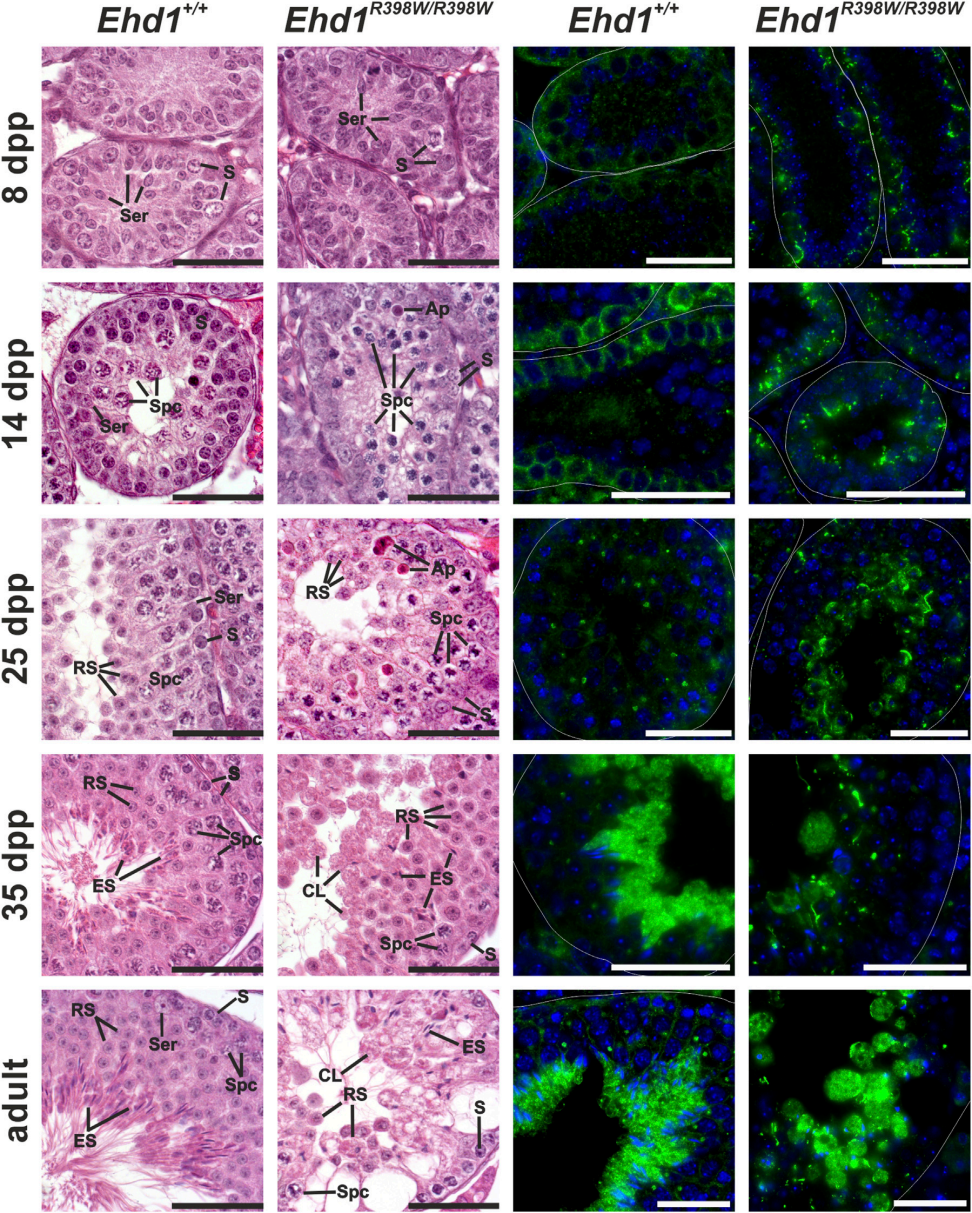
Spermatogenic failure in young knockin mice. Histological hematoxylin/eosin and immunofluorescence stains of Bouin-fixed, paraffin-embedded testicular tissue of young mice were performed to assess disturbances during the first spermatogenic wave. EHD1 protein expression was already altered 8 days after birth in mutant mice, whereas the first visible, histological differences were observed 14 days *postpartum*. *Ehd1* ^R398W/R398W^ mice showed an increased number of apoptotic cells (Ap) and elongated spermatid (ES) heads were misshaped and anchored deep within the epithelium instead of reaching for the luminal edge. Ser: Sertoli cell nucleus, S: spermatogonium, Spc: spermatocyte, Ap: apoptotic cell, RS: round spermatid, ES: elongated spermatid, CL: cytoplasmic lobe. Immunofluorescence staining: Green: EHD1, blue: HOE33342 (nuclei). Scale bar: 50 µm.

The massive extent of destruction of the seminiferous epithelium in adult *Ehd1* ^*R398W/R398W*^ mice made it impossible to identify early aberrant processes and to trace the underlying causes. To overcome this problem and clarify the initial onset of spermatogenic failure due to the *Ehd1* mutation, we took a closer look at different postnatal time points (Figure 6 and Supplementary Figure S5–S9). Within the first 35 days *postpartum* (dpp), we chose four time points (8, 14, 25, and 35 dpp) for more detailed insights into the apparent cell types, developmental progress, EHD1 protein expression pattern, and hints for initial disturbances.

Transverse sections of testicular tubules from 8-day-old wildtype mice contained mainly spermatogonia located at the basement membrane and Sertoli cells that built up the lumenless epithelium. Mitotic divisions were occasionally observed, and no obvious histological differences were evident between wildtype and mutant mice. However, localization and appearance of the EHD1 protein differed already at this early stage: although both genotypes showed some EHD1 protein near the spermatogonial stem cells close to the basement membrane, the shape of the EHD1-positive structures in *Ehd1* ^*R398W/R398W*^ mice differed from the regularly distributed wildtype EHD1 and the mutant protein appeared to form tubular aggregates that were absent in wildtype mice. 14 days *postpartum*, the first spermatocytes developed from the spermatogonia. Initial variations in cell morphology were present between the two genotypes. While *Ehd1* ^*+/+*^ mice had normal spermatocytes with the characteristic loose chromatin, spermatocytes from *Ehd1* ^*R398W/R398W*^ mice were smaller and had much denser chromatin, suggesting delay in the maturation process. EHD1 protein expression increased in both genotypes, but the localization of mutant EHD1 aggregates shifted slightly toward the luminal region. In contrast, wildtype EHD1 remained mostly basal (Figure 6 and Supplementary Figure S8).

By postnatal day 35, the first spermatogenic wave is completed (Oakberg, 1956). By this time, the seminiferous epithelium of wildtype mice showed the physiological ordered architecture consisting of a characteristic constellation of specific cell types. In the *Ehd1* ^*R398W/R398W*^ testes, assignment to specific spermatogenesis stages was not possible because the architecture of the epithelium was severely disturbed and the typical cell arrangement for each stage was not clearly identifiable (Figure 6 and Supplementary Figure S9). In addition, various abnormalities were observed, such as an unbalanced ratio of cell types, disturbances in chromatin condensation, and malformation of the elongating spermatid heads. Some of them were “trapped” deep in the epithelium instead of finding their way to the lumen. Membrane remodeling during spermatid maturation failed due to insufficient deposition of excess cytoplasm. Instead, huge, round vacuoles of cytoplasm and cell debris were deposited in the lumen of the tubules.

### Ultrastructural changes due to the EHD1 mutation

**FIGURE 7:**
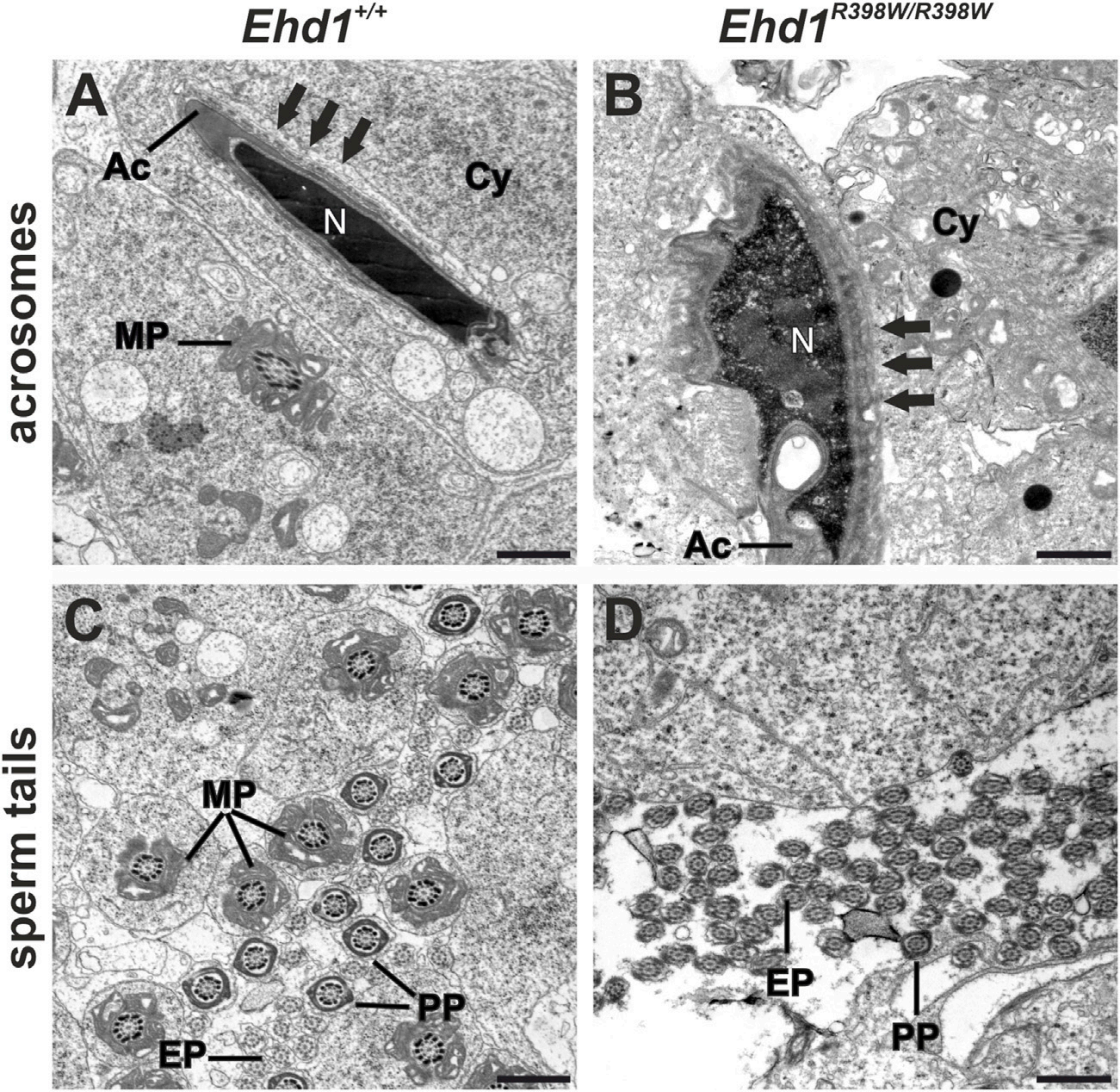
Electron micrographs of acrosomes and sperm tails. **(A)** *Ehd1* ^+/+^ mice showed normal-shaped heads with condensed chromatin in the nuclei (N) covered by the acrosome (Ac) exhibiting the typical dashed appearance of apical ectoplasmic specializations (arrows). Sperm tail midpiece cross-sections (MP) were observed in the cytoplasm (Cy). **(B)** A malformed acrosome (Ac) engulfed the misshaped head of an elongating spermatid displaying impaired chromatin condensation of the nucleus (N) and thickened ectoplasmic specializations (arrows) in *Ehd1* ^R398W/R398W^ mice. Additionally, the cytoplasm (Cy) appeared loosened and exhibited electron-dense droplets, probably lipid-droplets. **(C)** In wildtype mice, we found a high amount of cross-sections of sperm tail midpieces (MP), principal pieces (PP) and end pieces (EP), whereas the number of normal shaped midpieces was strongly decreased in *Ehd1* ^R398W/R398W^ mice **(D)**. Scale bars: 1 µm.

It is known that numerous processes in the sperm epithelium are at least partially dependent on endocytosis. We focused on acrosomal biogenesis and sperm tail assembly and aimed to investigate the effects of the *Ehd1* mutation on these processes at the ultrastructural level using transmission electron microscopy (TEM) (Figure 7). In terms of its function as a “tool” for the sperm to penetrate the outer layers of the oocyte, the acrosome has great importance during fertilization. Therefore, it is understandable that an intact form and unrestricted functionality is essential for reproduction. During the initial phase of acrosome biogenesis - the Golgi phase - active transport from the Golgi apparatus via the trans-Golgi network is essential for initiation of acrosome biogenesis (Berruti and Paiardi, 2011). Later, continuous transport of new material is also required during cap phase to “feed” the growing acrosome. Towards the acrosome and maturation phase, further growth becomes secondary, but extensive structural and membranous changes take place to generate the typical shape (Khawar et al., 2019). EHD1, as a protein known to be involved in endocytic vesicle transport and membrane shaping processes, appears to have a very specific function in this.

The longitudinal section of the wildtype spermatid showed the elongated head of a maturing spermatid with a well-condensed nuclear chromatin (N) covered by the sickle-shaped acrosome (Figure 7A). This sperm precursor was anchored in the epithelium via apical so-called ectoplasmic specializations (ES; arrows in Figure 7A), a testis-specific, actin-based, tight junction-like anchorage composed of actin filament bundles and endoplasmic reticulum cisternae (Yan et al., 2007; Wan et al., 2013). The connection between apical ectoplasmic specializations and elongated spermatids was visible as a dashed line. This anchoring of spermatids is essential for proper sperm maturation. However, when ectoplasmic specializations degradation is dysfunctional, this may cause severe problems as spermatids cannot detach from the epithelium. Ectoplasmic specializations occur not only apically but also in the basal compartment. Here, the basal ectoplasmic specializations assume a very important function, being one of the key components of the blood-testis barrier. In contrast to the wildtype, the vast majority of *Ehd1* ^*R398W/R398W*^ sperm heads showed incomplete chromatin condensation in combination with nuclear and acrosomal malformations, thickened ectoplasmic specializations with irregular boundaries, and a cytoplasm containing numerous vacuoles and putative lipid droplets (jet-black round structures).

**FIGURE 8:**
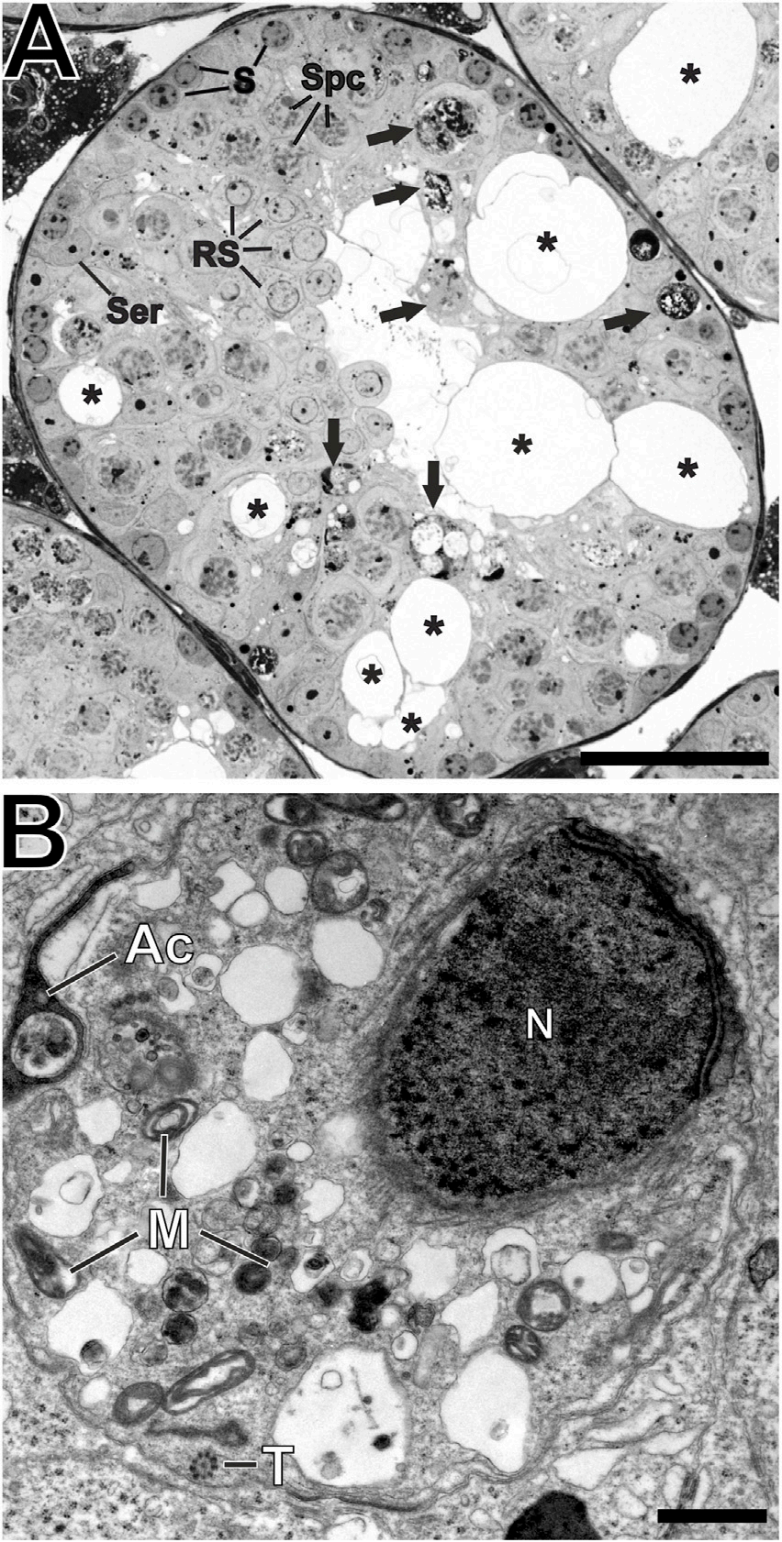
Destroyed architecture of seminiferous tubules. **(A)** Richardson-stained semi-thin section of Epon-embedded adult testicular tissue showing the perforated epithelium (asterisks) of *Ehd1* ^R398W/R398W^ mice hosting different cell types like spermatogonia (S), spermatocytes (Spc), round spermatids (RS) and Sertoli cell nuclei (Ser). However, these cells showed various malformations and the composition of cells could not be clearly addressed to any of the 12 defined spermatogenesis stages. Damaged or malformed cellular components are stored in round, membranous structures (black arrows) and phagocytosed by Sertoli cells. Scale bar: 50 µm. **(B)** A higher magnification of these round, membranous structures revealed its content: Defective parts of malformed sperm precursors like spermatid head with loose nuclear chromatin (N), misshaped acrosomal-derived structures (Ac), as well as damaged mitochondria (M) and sperm tail axonemes (T). Scale bar: 500 nm.

Each part of the sperm tail is characterized by additional structures that ultimately ensure proper tail beating: characteristic of the midpiece are 1) the mitochondrial sheath that provides the energy supply for motility, 2) 9 outer dense fibers that support wave formation, and 3) an axoneme - the core structure - consisting of 9 microtubule duplicates and a central pair of microtubules with dynein arms and radial spokes. The principal piece lacks the mitochondrial sheath and is characterized by circumferential ribs of the fibrous sheath, which are working together with outer dense fibers to support the wavy tail beat, while the microtubule core remains the same as in the midpiece. The end piece has a smaller diameter and lacks accessory structures (Lehti and Sironen, 2017). In seminiferous tubules of wildtype mice, numerous cross-sections of all three parts of sperm tails with the characteristic accessory structures were observed in the luminal region of the sperm epithelium (Figure 7). In *Ehd1* ^*R398W/R398W*^ we consistently found sperm tail cross-sections of the principal and end pieces that had a normal 9x2+2 axonemal structure, indicating that ciliogenesis itself is unaffected by the mutation. However, the number of midpieces with the characteristic mitochondrial sheath was strongly decreased in knockin mice (Figure 7D). Midpieces were also virtually absent in overview images (Richardson staining) of *Ehd1* ^*R398W/R398W*^ but clearly detectable in *Ehd1* ^*+/+*^ mice (Supplementary Figure S11, S12). In *Ehd1* ^*R398W/R398W*^ tissue, remnants of midpieces and clusters of damaged mitochondria were observed in large vacuoles likely representing autophagosomes and autolysosomes (Figure 8).

**FIGURE 9:**
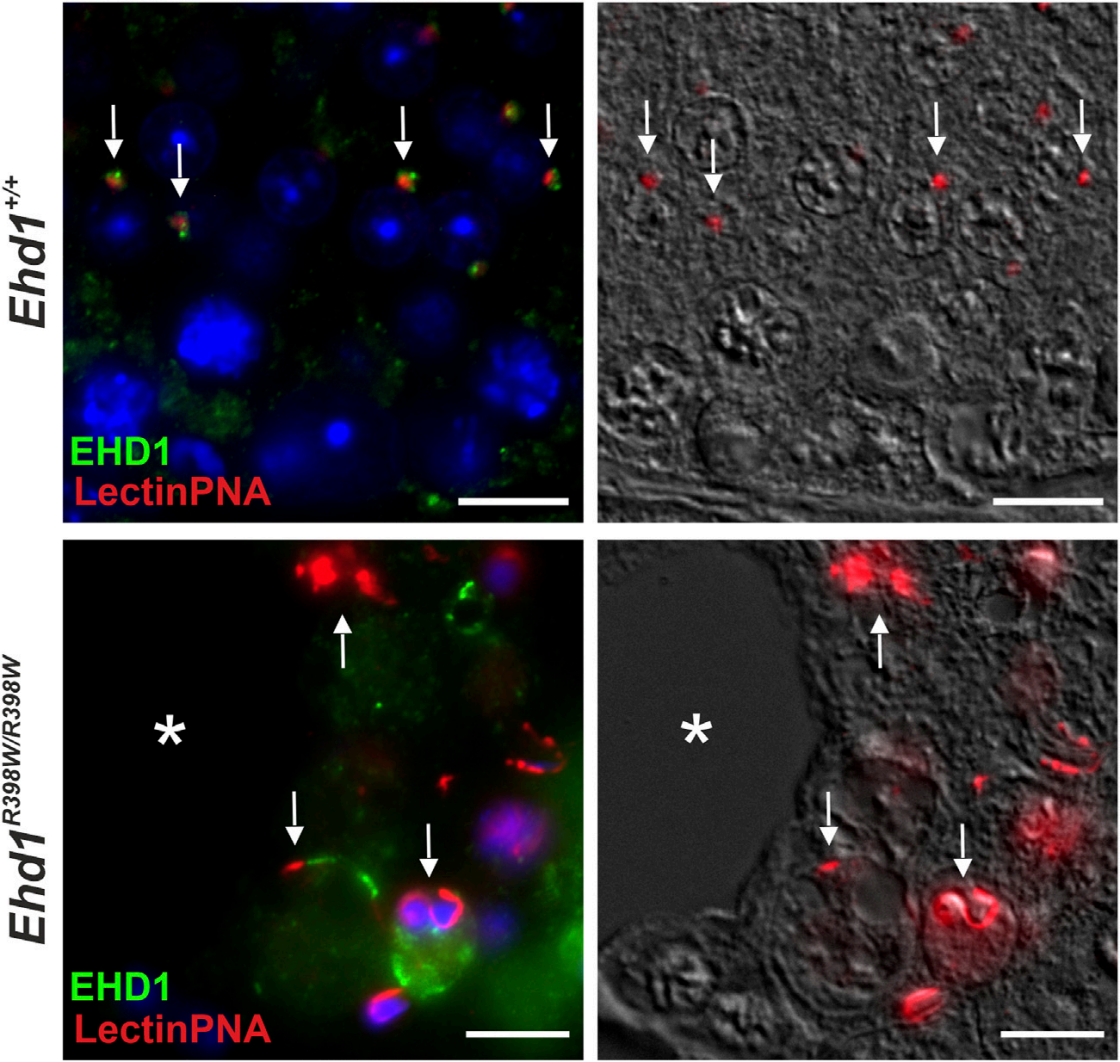
Mutant EHD1 was unable to interact with acrosomal granules in mouse testis. Wildtype EHD1 co-localized to acrosomal granules on round spermatids suggesting an important function in acrosomal biogenesis. In contrast, mutated EHD1 did not interact with these structures resulting in severe acrosomal malformations. Green: EHD1, red: LectinPNA (acrosome), blue: HOE33342 (nuclei). Scale bar: 10 µm.

The presumed association between EHD1 and acrosome formation was confirmed by immunofluorescence (Figure 9). In *Ehd1* ^*+/+*^ mice, EHD1 was clearly colocalized with early acrosomal granules visualized by LectinPNA staining. EHD1-positive spots appeared to surround the red-stained vesicles at one pole of the round spermatids. These granules will continue to increase in size and become sickle-shaped by continuous supply of material from the Golgi and plasma membrane through endocytic transport processes. In contrast, such co-localizations between acrosomal granules and mutant EHD1 were almost completely absent in the seminiferous epithelium of *Ehd1* ^*R398W/R398W*^ mice.

### Impaired interaction of the mutant EHD1 with the retromer

Initial links between EHD1 and retromer were discovered several years ago (Zhang et al., 2012a). It is known that retrograde transport from endosomes to the trans-Golgi network occurs through a variety of regulators, with the retromer being one of the most important. It is also known that EHD1 interacts with the retromer components VPS26 and VPS35. However, the detailed mechanisms of this interaction and its relevance as well as other factors involved are not yet known (Zhang et al., 2012a). In the testis, the retromer has not been studied in detail and little is known about its function during spermatogenesis. The retromer components VPS26A and VPS35 have previously been shown to interact with early and late endosomes as well as acrosomal structures in haploid male germ cells, suggesting a potential role for retromer in acrosomal biogenesis (Da Ros et al., 2015). Given the acrosomal malformations in the presence of the mutant EHD1, it would be conceivable that impaired interaction of EHD1 with the retromer contributes to this phenotype.

**FIGURE 10:**
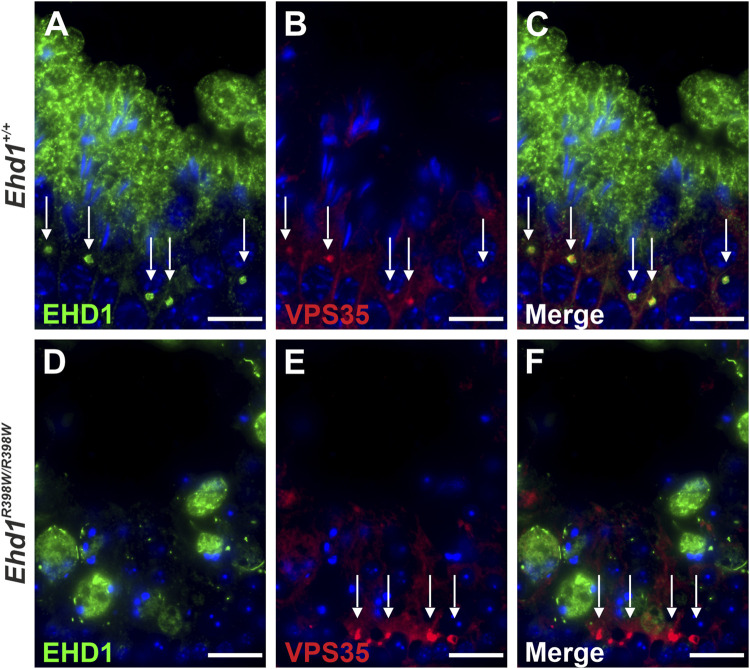
Localization of EHD1 and the retromer component VPS35 (white arrows) in mouse testis. While wildtype EHD1 was observed to engulf VPS35-positive vesicles located in the cytoplasm of spermatocytes (upper panel), this assembly was absent in *Ehd1* knockin mice (lower panel). Instead, the VPS35-positive vesicles built aggregates in near proximity to spermatogonial stem cells and the blood-testis barrier region. Green: EHD1, red: VPS35, blue: HOE33342 (nuclei). Scale bar: 10 µm.

We examined the localization of VPS35-positive vesicles in the seminiferous epithelium and discovered a distinct co-localization of the retromer and EHD1 in close proximity to spermatocytes (Figure 10). Wildtype EHD1 appeared to encapsulate the retromer component VPS35, whereas mutant EHD1 was altered in shape and localization, being mainly visible in large cytoplasmic lobes but not close to VPS35. In *Ehd1* ^*R398W/R398W*^ mice, the retromer complex appeared to be stuck near the basement membrane. These data suggest that the mutation impaired the ability of EHD1 to regulate retromer function, leading to severe disruption of spermatogenesis.

**FIGURE 11:**
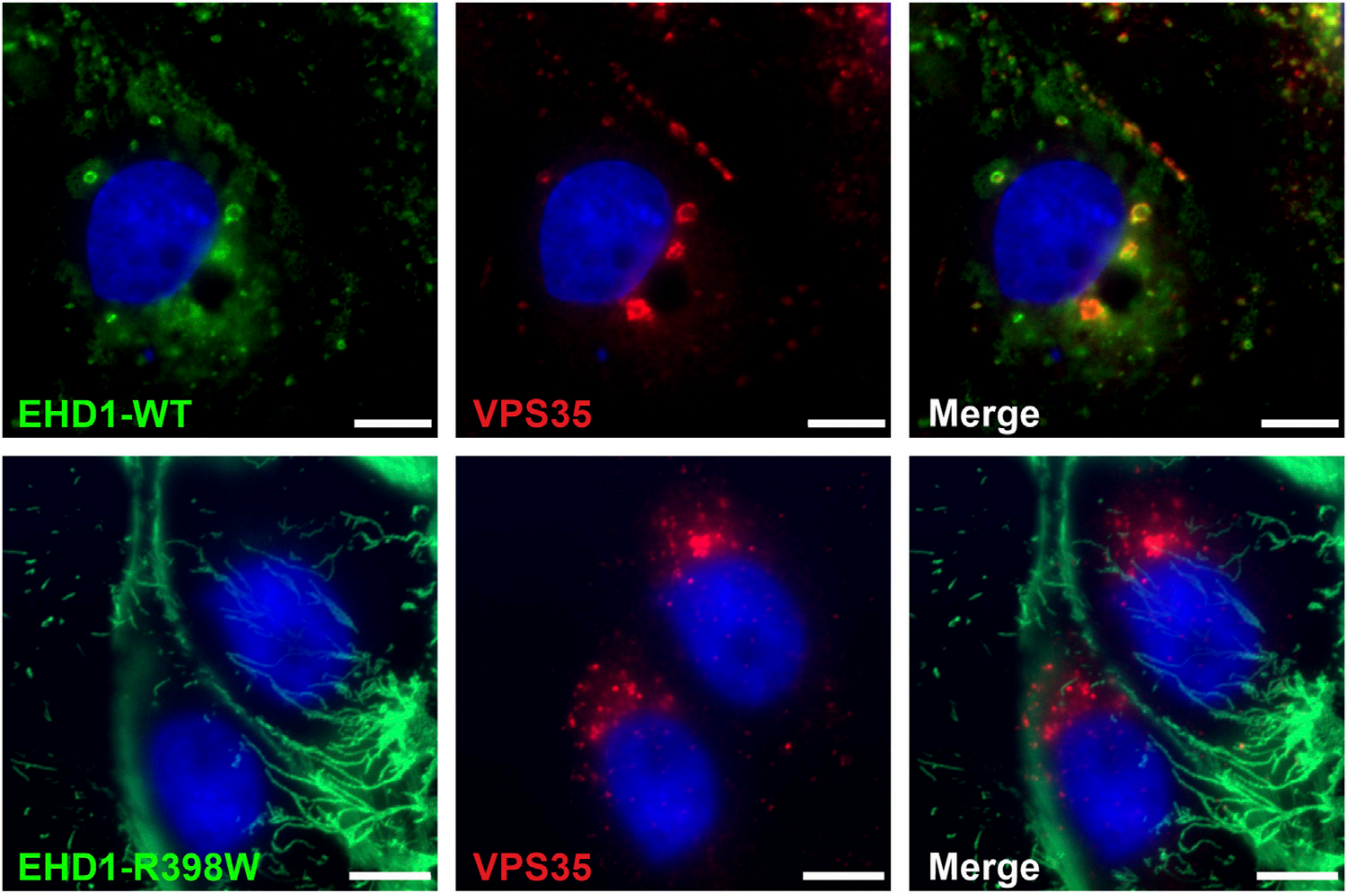
Mutant EHD1 fails to interact with VPS35 in LLC-PK1 cells. Inducible LLC-PK1 cells either expressed the wildtype or mutant form of human EHD1 upon tetracycline treatment. While wildtype EHD1 showed a well-distributed, dotted appearance and clearly co-localized to VPS-positive vesicles (upper panel), mutated EHD1 was observed in long, tubular structures that did not show any interaction with the retromer (lower panel). Green: EHD1, red: VPS35 (retromer), blue: HOE33342 (nuclei). Scale bar: 10 µ.m.

Complementing the interaction of EHD1 and the retromer in testicular tissue, we wanted to test whether these results can be reproduced in a cell model. LLC-PK1 cells (porcine proximal tubular cells) were genetically modified to overexpress either wildtype or mutant form of human EHD1 protein after tetracycline induction. Whereas LLC-PK1 cells expressing wildtype EHD1 showed a punctate, uniform distribution of the protein in the cytoplasm, the mutant protein was detected in elongated, tubular structures (Figure 11). These mutant EHD1-positive tubular structures are thought to be the result of impaired membrane cleavage due to defective EHD1 function (Issler et al., 2022). Regarding the interaction of EHD1 and the retromer, results were similar to those observed in testicular tissue: whereas wildtype EHD1-containing vesicles colocalized with the retromer and EHD1 appeared to engulf the retromer, mutant EHD1 lost the ability to interact with VPS35. Instead, VPS35 appeared to accumulate in close proximity to the nucleus, while mutant EHD1 was observed in long tubes that extended throughout the cytoplasm.

## Discussion

EH domain-containing protein 1 (EHD1) is part of a protein complex that orchestrates intracellular membrane organization and vesicular scission. It is required for ciliogenesis in certain cell types (Lu et al., 2015; Bhattacharyya et al., 2016; Lu and Westlake, 2021). Recently, we described the first human disease caused by a mutation in EHD1: The homozygous p.R398W missense mutation of EHD1 resulted in tubular proteinuria and sensorineural hearing loss in affected patients (Issler et al., 2022). In addition to the kidney and inner ear, EHD1 is highly expressed in the testis. Male *Ehd1* knockout mice exhibit marked pathological changes in the testes and are infertile (Rainey et al., 2010). Male *Ehd1* ^*R398W/R398W*^ mice are also infertile (Issler et al., 2022), but it was not known whether the p.R398W missense mutation affects testicular function in the same way as inactivation of the *Ehd1* gene. Therefore, in this study, we examined the testicular phenotype in an *Ehd1* ^*R398W/R398W*^ knockin mouse in detail. Spermatogenesis of *Ehd1* ^*R398W/R398W*^ mice was disrupted at multiple levels: 1) testes were smaller in size; 2) normally shaped spermatozoa were virtually absent in the testes and epididymides; 3) the formation of the acrosome and the midpiece of the sperm tail were disturbed; 4) the seminiferous tubules had large vacuoles, their well-organized cellular architecture was completely disrupted, and the different physiological stages of spermatogenesis could no longer be distinguished. The severe testicular phenotype and infertility of male *Ehd1* ^*R398W/R398W*^ mice suggests that *EHD1* may also be a candidate gene for male infertility in humans.

## Physiology and pathophysiology of EHD1

EHD1 is an important component of a complex machinery that shapes intracellular membranes and participates in the transfer of membranes, receptors, and cargo by pinching off vesicles. The importance of intracellular membrane and vesicle dynamics is evident from the variety of processes that may depend on EHD1, such as receptor recycling (Caplan et al., 2002; Rapaport et al., 2006), endocytosis (Mintz et al., 1999; Issler et al., 2022), retromer function (Zhang et al., 2012a; Dhawan et al., 2020), mitochondrial homeostasis (Farmer et al., 2017; Farmer et al., 2018), and ciliogenesis (Lu et al., 2015; Xie et al., 2019; Lu and Westlake, 2021). Numerous studies on EHD1 have been performed in cell cultures. Important insights into the *in vivo* relevance of EHD1 were provided by studies on *Ehd1* knockout mice. Interestingly, the published phenotypes of knockout mice vary considerably, ranging from very mild changes (Rapaport et al., 2006) to male infertility (Rainey et al., 2010), defective ocular lens development (Arya et al., 2015), and embryonic lethality (Bhattacharyya et al., 2016). Some of the differences were attributed to different genetic backgrounds of the mice (Bhattacharyya et al., 2016).

The relevance of EHD1 in humans is supported by a recently published study in which we found an association between a homozygous mutation of EHD1 (p.R398W) and a syndrome characterized by hearing loss and tubular proteinuria (Issler et al., 2022). At the cellular level, the p.R398W mutation resulted in defective scission of vesicles and the formation of pathologically long tubulovesicles (Issler et al., 2022). What is the pathomechanism of the p.R398W mutation of EHD1? In our previous study, we used an *in silico* structural biology approach to address this question (Issler et al., 2022). The simulations suggested that the p.R398W mutation affects the oligomerization of EHD1. Furthermore, it appeared conceivable that the mutation interferes with the ability of EHD1 to bind and hydrolyze ATP. Oligomerization and ATP hydrolysis are most likely a prerequisite for EHD1 to function as a membrane-forming protein in assisting scission of intracellular vesicles.

Also during spermatogenesis, the dynamics of intracellular membranes are crucial for a number of steps required for the development of germ cells into mature spermatozoa. Here, we used an *Ehd1* ^*R398W/R398W*^ mouse model to investigate whether this mutation leads to a testicular phenotype maybe similar to the one described for the knockout mouse.

## Expression and localization of EHD1 in mouse testis

EHD1 is highly expressed in testicular tissue (Mintz et al., 1999). In our study, EHD1 mRNA was found at high levels in seminiferous tubules at almost all stages of spermatogenesis (Figure 3), being low only at stages IX and X. It is also striking that mRNA expression was very high in meiotic cells and round spermatids, but very low in basal spermatogonia, elongated spermatids, and mature spermatozoa. Interestingly, protein expression differed markedly from mRNA expression in terms of localization and stage dependency: Protein expression was restricted to stages III-VII and was mainly found in elongated spermatids towards the lumen of seminiferous tubules (Figure 3).

How can the discrepancy between mRNA and protein expression be explained? It is known that the differentiation processes during spermiogenesis are associated with extensive changes in the nucleus, including chromatin condensation of the developing spermatids. The associated replacement of histones by protamines impairs the accessibility of the transcriptional machinery to DNA and leads to transcriptional arrest. Accordingly, much of the mRNAs of a variety of genes is produced earlier during the transcriptionally active phase of round spermatids but is not translated until later stages of spermatogenesis, i.e., so-called ‘translational delay’ (Kleene, 1996; Braun, 1998; Kleene, 2016). The data presented here suggest that also EHD1 expression appears to be subject to translational delay.

### Testis phenotype of *Ehd1* ^*R398W/R398W*^ mice

Male *Ehd1* knockin mice were infertile (Issler et al., 2022), a phenotype reminiscent of the testis phenotype of *Ehd1* knockout mice (Rainey et al., 2010). The testes and epididymides of the knockin mice were significantly smaller than those of age-matched wildtype animals (Figure 2). On the microscopic level, the marked destruction of the normal structure of the seminiferous tubules made it challenging to identify the underlying mechanisms. Nevertheless, based on the studies performed here, it can be assumed that the p.R398W mutation of EHD1 leads to a mislocalization of the mutant protein and profoundly affects various steps of physiological spermatogenesis.

- Impaired spermiogenesis

Both, *Ehd1* ^*−/−*^ *and Ehd1* ^*R398W/R398W*^ mice were unable to produce offspring and no mature sperm were found in the epididymis. The absence of mature sperm was attributed to a failure of spermiogenesis (Rainey et al., 2010; Issler et al., 2022). Also in *Ehd1* ^*R398W/R398W*^ mice, the major problems occurred during the maturation process from meiotic spermatocytes to round spermatids and later to elongating spermatids, ultimately leading to decreased production of mature, functional sperm and increased phagocytosis of cellular debris. Most likely, the massive damage of the testicular epithelium led to progressive atrophy of large tubular segments and reduced size of the testes compared to wildtype mice. The severity of pathology in *Ehd1* knockin and knockout mice is indicative of insufficient compensatory mechanisms, perhaps because expression of other members of the EHD family in testicular tissue is too low or the different EHD proteins have distinct functions. In addition, phenotypic similarities between *Ehd1* knockin and knockout mice suggest that the p.R398W mutation may elicit its pathological consequences via a loss-of-function mechanism. However, we cannot rule out the possibility of an additional gain-of-function component caused by the mislocalized mutant EHD1 protein (Figure 4). Moreover, it is yet unclear whether EHD1 has a specific function at the head-to-tail coupling apparatus (HTCA), a structure connecting the sperm head and the tail (Shang et al., 2017; Tapia Contreras and Hoyer-Fender, 2019; Wu et al., 2020) (Figure 5).

- Disturbed acrosome formation and retromer composition

EHD1 is a key component of the intracellular membrane shaping machinery that allows budding and dynamics of vesicles. Therefore, it is not surprising that functional impairment of EHD1 by genetic inactivation or by a deleterious missense mutation impacts on cellular processes that involve intracellular vesicles. The acrosome is a unique large vesicle covering the anterior part of the sperm nucleus. Similar to lysosomes, the acidic acrosome contains a number of hydrolytic enzymes that are needed for the penetration of the sperm through the zona pellucida of the egg. Acrosome biogenesis is still incompletely understood but it involves the endosomal pathway and Golgi complex-derived vesicle trafficking including retromer-dependent processes (Berruti and Paiardi, 2011; Da Ros et al., 2015). The retromer complex is known to play an important role in the highly complex and multilayered endocytosis machinery as it regulates the distribution of cargo by retrograde trafficking (McKenzie et al., 2012). The retromer is also thought to play an important role in autophagy (Cui et al., 2019), in the lysosomal system (Maruzs et al., 2015), in mitochondrial dynamics (Farmer et al., 2017) and interacts with RNA granules in haploid germ cells (Da Ros et al., 2015). An interaction between retromer components and EHD1 has already been described, although the precise mechanisms remain elusive (Gokool et al., 2007; Farmer et al., 2017). Interestingly, mutant EHD1 lost the ability to interact with the retromer complex in testicular tissue and in an overexpressing LLC-PK1 cells model (Figures 10, 11). Probably caused by impaired dynamics of intracellular vesicles and disturbed retromer function, acrosomal malformations were present in *Ehd1* knockin (Figure 7) and knockout mice (Rainey et al., 2010). In addition, *Ehd1* ^*R398W/R398W*^ mice showed large, round structures in the testicular epithelium with entrapped defective or partially degraded material. It is conceivable that these large vacuoles are autophagosomes or autolysosomes. In this context, it is interesting to note that MICAL-L1 and pacsin-2, two EHD1-associated proteins, remain associated even with the mutant EHD1 protein (Issler et al., 2022). VPS35, on the other hand, did not colocalize with the mutant protein (Figures 10, 11). This may argue that VPS35 and EHD1 do not interact directly. The relevance of the EHD1/retromer interaction in the context of spermatogenesis and acrosome formation, the conditions required for an interaction and other factors involved remain to be investigated in future studies.

- Impaired assembly of sperm tails

The sperm tail is a complex structure that is essential for directed sperm locomotion and belongs to the class of motile cilia. During the development of the flagellum, complex membrane remodeling and transport processes play a critical role. EHD1 can have an essential role in the development of primary cilia (Lu et al., 2015; Bhattacharyya et al., 2016; Jones et al., 2022), so it is conceivable that it is also required for sperm tail formation. Interestingly, from patients carrying the p.R398W mutation, no symptoms indicating a ciliopathy were reported (Issler et al., 2022). Here, we used electron microscopy to investigate the ultrastructure of sperm tails. Since the formation of the characteristic flagellar/ciliary structure with the 9x2+2 pattern of microtubules was preserved in knockin mice, sperm ciliogenesis *per se* appeared unaffected, but the relocalization of cytoplasmic mitochondria to the tail midpiece during sperm elongation was disturbed. Cross sections of normally structured mid-pieces of sperm tails were strongly decreased in *Ehd1* ^*R398W/R398W*^ mice (Figure 7 and Supplementary Figure S10. S11). Instead, remnants of midpieces were found together with other material in large vacuoles that most likely corresponded to autophagosomes and autolysosomes (Figures 7, 8). Interestingly, there is growing evidence that EHD1 and components of the retromer could affect mitochondrial homeostasis and interorganellar crosstalk (Farmer et al., 2017; Farmer et al., 2018; Santos et al., 2022). Such mechanisms might contribute to the apparently defective assembly of the midpiece.

- Disturbed tubular architecture and leaky blood-testis-barrier

In mammals, the blood-testis barrier serves to protect the maturing cells from “external influences” that potentially pose a threat. Localized between two adjacent Sertoli cells, it is not a static structure, but subject to constant remodeling (opening and closing) in order to manage the transit of preleptotene spermatocytes from the basal to the adluminal compartment during late stage VII to VIII of spermatogenesis (Cheng et al., 2011). This gating is known to be based on endocytosis, transcytosis, and endosome-mediated degradation and recycling processes (Cheng and Mruk, 2012). Interestingly, mRNA and protein expression of EHD1 was highest immediately prior to these events and decreased immediately afterwards, suggesting a role of EHD1 in this process. In adult *Ehd1* ^*R398W/R398W*^ knockin mice, the sophisticated architecture of the testicular tubules was severely disrupted and the precise localization of the different cell types and cell stages within the epithelium was abolished. It can therefore be assumed that the blood-testis barrier was also no longer intact. However, it is hardly possible to find out whether a disturbance in the structure of the blood-testis barrier contributes to the development of the disturbed tubule architecture or is a consequence of it.

## EDH1: Relevance for male infertility in humans?

We recently described the clinical phenotype of patients homozygous for the EHD1 mutation R398W. These patients live in a crisis region of the Middle East and we no longer have access to them. Therefore, we have not been able to directly determine whether the R398W mutation also causes a testicular phenotype or male infertility in humans. Is there any other evidence that EHD1 might also be relevant to male fertility in humans? A clear pathophysiological link has not yet been established in males, but circumstantial evidence supports a role for EHD1 in spermatogenesis (http://mik.bicnirrh.res.in/search81.php?Gene_Symbol=10938):

EHD1 is one of many sperm-derived RNAs that were reduced in sperm from teratozoospermic individuals (supplementary information of (Platts et al., 2007)). In a genomic screen for possible candidates responsible for discordant cryptorchidism in monozygotic twins, the EHD1 gene was one of the differentially methylated genes (supplementary information of (Lu et al., 2017)). In another study, EHD1 was identified as a downregulated factor associated with postmeiotic and meiotic arrest of spermatogenesis (supplementary information of (Kui et al., 2019)).

In addition, the human EHD1 gene is highly likely to be loss-of-function intolerant (pLI) (https://gnomad.broadinstitute.org/gene/ENSG00000110047?dataset=gnomad_r2_1). Virtually all severely haploinsufficient human disease genes are loss-of-function intolerant, but EHD1 does not appear to be haploinsufficient because heterozygous individuals do not show pathology (Issler et al., 2022). Another reason for a high probability of being loss-of-function intolerant is a reproductive disadvantage (Lek et al., 2016), which may be the case for EDH1.

Taken together, the severe impairment of spermatogenesis in *Ehd1* ^*R398W/R398W*^ and *Ehd1* ^*−/−*^ mice and the circumstantial evidence for a role of EHD1 in human testis make *EHD1* a potential candidate gene for male infertility. Mutations in human *EHD1* should therefore be considered in cases of male infertility of unknown cause.

## Data Availability

The original contributions presented in the study are included in the article/supplementary material, further inquiries can be directed to the corresponding author.
