# Supplementary material for: A missense mutation in Ehd1 associated with defective spermatogenesis and male infertility
Source: Front Cell Dev Biol. 2023 Oct 12;11:1240558. doi: 10.3389/fcell.2023.1240558 (PMC10600459; doi:10.3389/fcell.2023.1240558)
Supplement: Supplementary file 1 [file DataSheet1.PDF]

Supplementary Material

A missense mutation in *Ehd1* associated with defective spermatogenesis and male infertility

Katrin Meindl [1], Naomi Issler [2,3], Sara Afonso [1,4], Alberto Cebrian-Serrano [5,6,7], Karin Müller [8] Christina Sterner [1], Helga Othmen [1,9], Ines Tegtmeier [1], Ralph Witzgall [9], Enriko Klootwijk [2], Benjamin Davies [5,10], Robert Kleta [2], Richard Warth [1,*]

[1] Medical Cell Biology, University Regensburg, Germany

[2] Department of Renal Medicine, UCL, London, UK

[3] Pediatric Nephrology Unit and Research Lab, Hadassah Medical Center and Faculty of Medicine, Hebrew University of Jerusalem, Jerusalem, Israel

[4] Institute of Cellular and Molecular Physiology, Friedrich-Alexander-Universität Erlangen-Nürnberg, Germany

[5] Wellcome Centre Human Genetics, University Oxford, UK

[6] Helmholtz Zentrum München, Institute of Diabetes and Obesity, Munich, Germany

[7] German Center for Diabetes Research (DZD), Neuherberg, Germany

[8] Leibniz Institute for Zoo- und Wildlife Research, Berlin, Germany

[9] Molecular and Cellular Anatomy, University Regensburg, Germany

[10] Genetic Modification Service, The Francis Crick Institute, London, UK.

SUPPLEMENTARY FIGURES

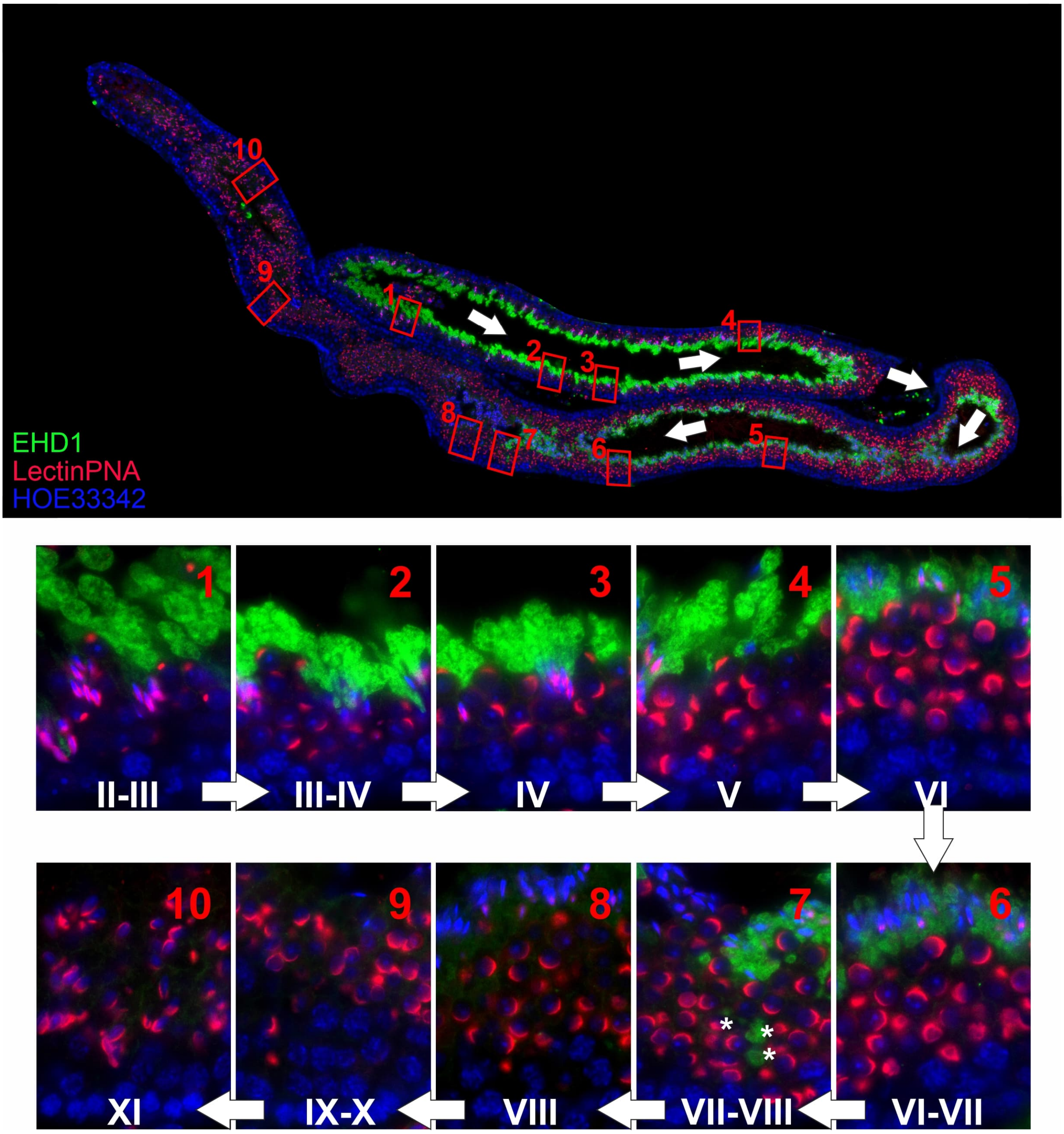

SUPPLEMENTARY FIGURE 1: Spermatogenic wave.

An isolated, Bouin-fixed and paraffin-embedded testicular tubule was sectioned longitudinally to examine the EHD1 expression pattern during the course of spermatogenesis. 10 sections (1-10) were magnified and the tubular stages were indicated by Roman numerals. EHD1 appeared in a stage-dependent manner: From stage II-IV, expression increased steadily and finally concentrated to the

region of elongated spermatids lining up at the tubule lumen in V-VI. After successful detachment of spermatozoa from the epithelium in late stage VII and early stage VIII, the EHD1 signal vanished. Prior to spermiation, excess cytoplasm was shed from mature spermatids and was phagocytosed by Sertoli cells. These residual bodies also appeared to contain EHD1 and were visible in the seminiferous epithelium at stage VII-VIII (asterisks). Green: EHD1; red: LectinPNA (acrosomes); blue: HOE33342 (nuclei).

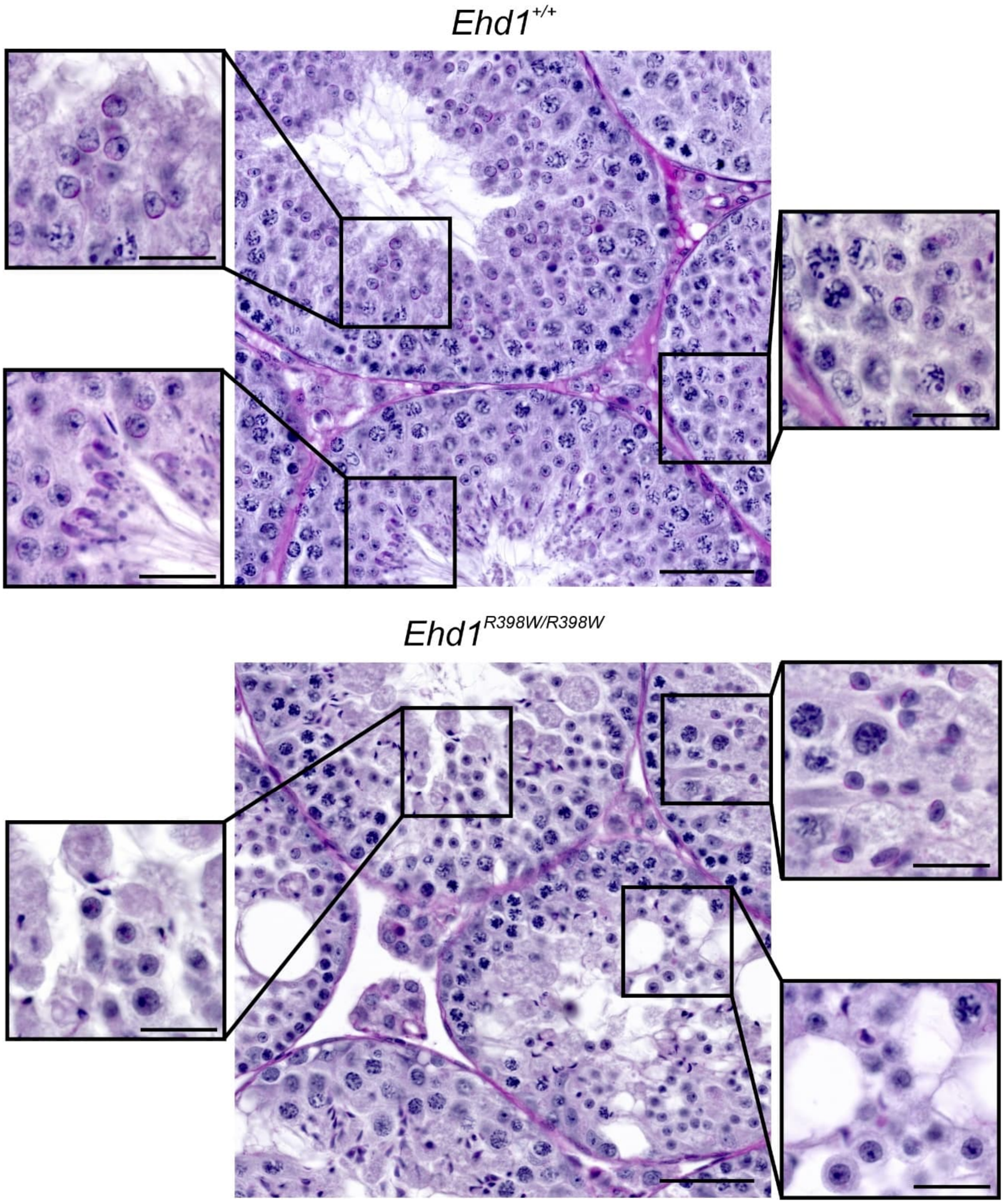

SUPPLEMENTARY FIGURE 2. PAS stain of testes of an adult wildtype and an adult *Ehd1*^{R398WR398W} mouse

Please not the severe disruption of the normal tubular architecture in knockin mice. Bars 50 µm (overview images) and 20 µm (magnified images). Periodic acid Schiff (PAS) stain was performed on 5 µm thick paraffin sections. After deparaffinization and rehydration twice in xylene, 99%

isopropanol, and decreasing concentrations of ethanol (95%, 80%, 70%) for 12 minutes each, sections were washed in aqua dest 3 x 2min. Sections were further incubated in 0.5% periodic acid for 10 min before they were washed 3 x 2 min in tap water. After that, the sections were placed in Schiff's reagent (Sigma Aldrich) for 15 min at room temperature. To avoid pseudo-PAS staining, they were washed 3 x 2 min in fresh 0.5% sulphite water. To remove the sulphite water, they were incubated in tap water for 10 min and then in distilled water for 3 x 2 min, followed by co-staining with haemalaun for 1 min. The sections were blued in running tap water for 10 min, followed by short incubation (3 sec) in ascending EtOH series and xylene (2 x 5 min). The slides were mounted in 223 DePeX mounting medium (Serva Electrophoresis GmbH, Heidelberg, Germany).

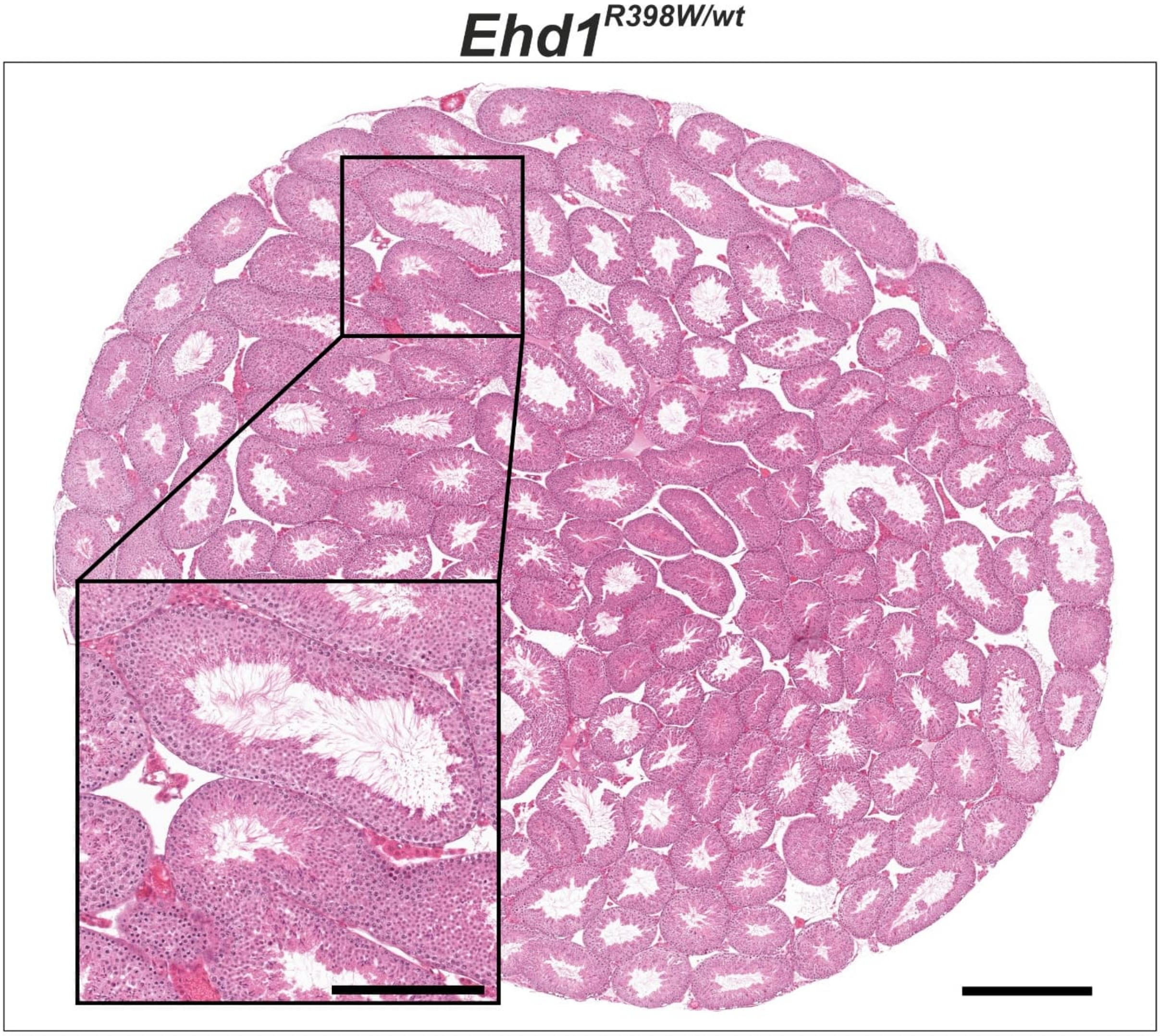

SUPPLEMENTARY FIGURE 3. Testis histology of a heterozygous *Ehd1*$^{R398W/wt}$ mouse

Heterozygous, male Ehd1$^{R398W/wt}$ *mice were fertile as they were able to produce offspring. In order to see if the mutation of one allele was sufficient to cause impairments in spermatogenesis, these mice were also examined: The epithelium appeared comparably homogeneous to that of wild-type mice and no obvious defects could be detected. The stages of spermatogenesis were easily identified and germ cell maturation did not appear to be affected. Accordingly, the mutation seems to affect fertility only in the homozygous state. Scale bar: 250µm (magnified image) / 500µm (overview image).*

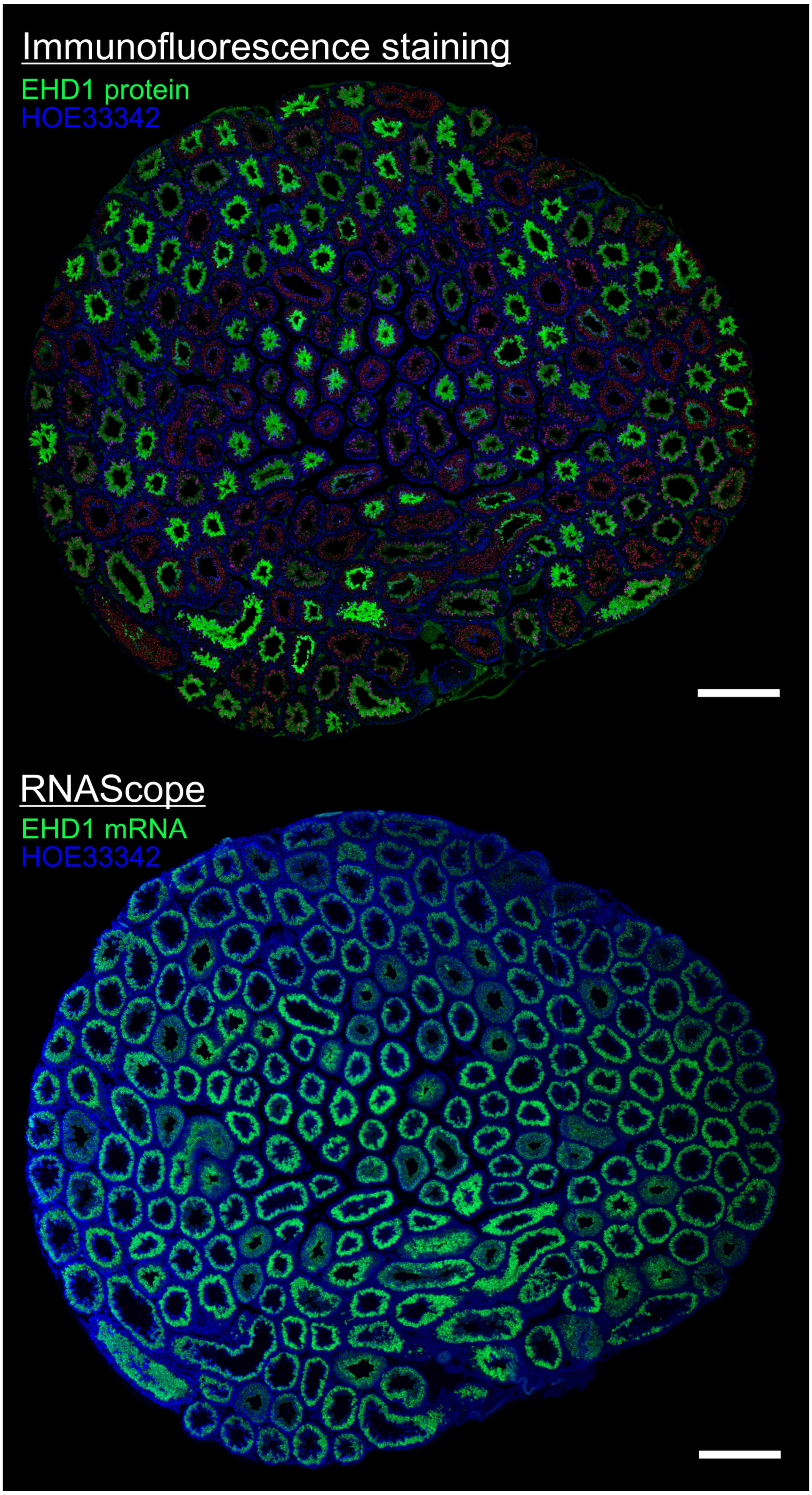
Immunofluorescence staining
EHD1 protein
HOE33342
RNAScope
EHD1 mRNA
HOE33342

SUPPLEMENTARY FIGURE 4. Overview images of EHD1 mRNA and protein.

Cross-sectional images of consecutive sections of a wildtype testis depict the expression of EHD1 protein (IF) compared to EHD1 mRNA (RNAScope). While protein exhibited a strong stage-dependent expression pattern, the stage dependence was less pronounced for mRNA expression. The localization within the seminiferous epithelium also differed, with the protein being predominantly luminal, whereas the mRNA was detected centrally within the epithelium. This temporal and spatial discrepancy is probably due to the translational delay experienced by maturing germ cells, as the increasingly condensed nucleus of elongating spermatids does not allow further transcriptional events. As a result, the mRNA is produced at a time when the genome is easily accessible, but is not translated into protein until later. Green: EHD1 protein or mRNA; red: LectinPNA (acrosomes); blue: HOE33342 (nuclei). Scale bar: 500µm.

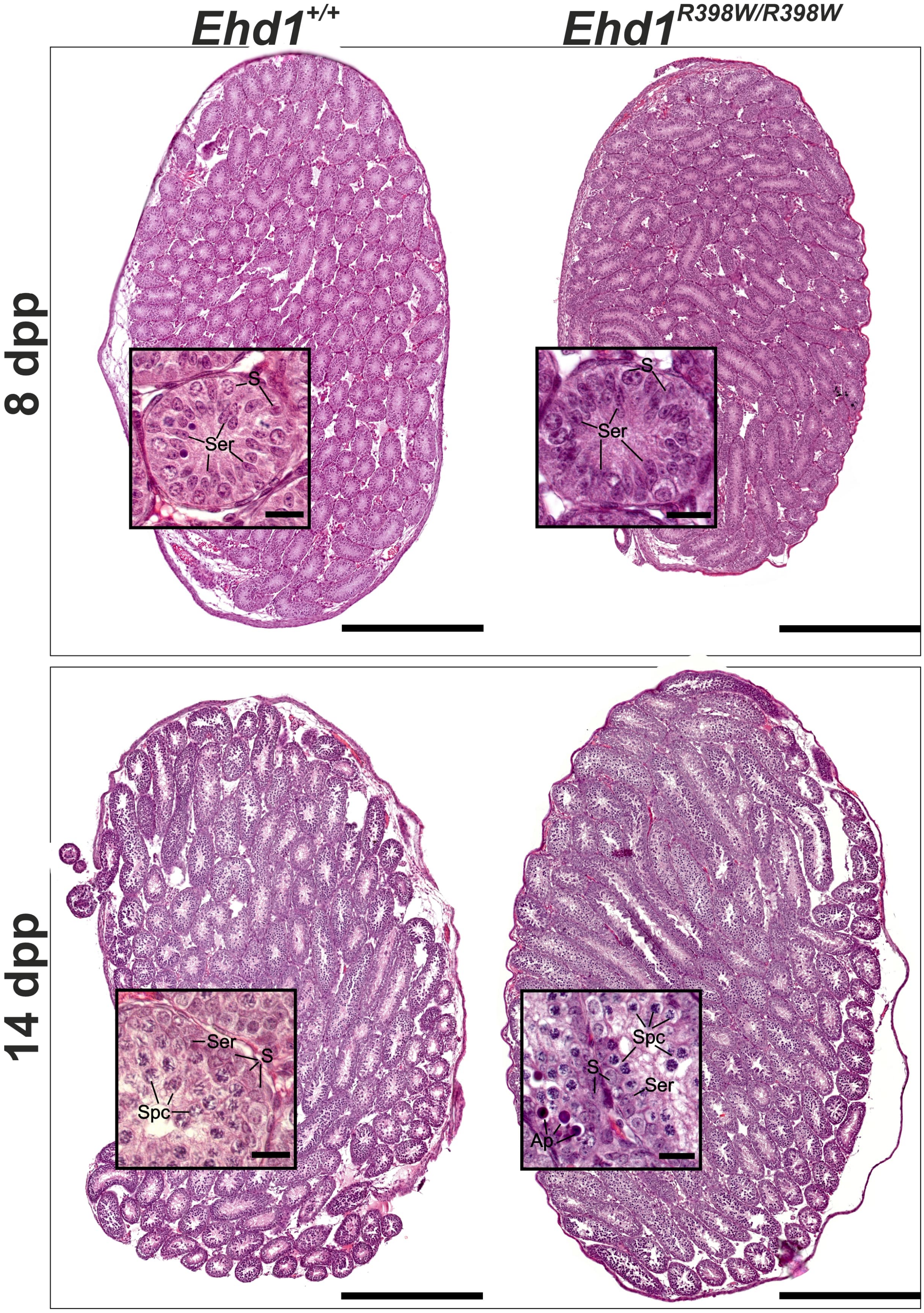
Ehd1+/+
Ehd1R398W/R398W
8 dpp
14 dpp
S
Ser
Spc
Ap

SUPPLEMENTARY FIGURE 5. Overview images of testes at 8 and 14 days postpartum

Overview HE stains of testes from 8- and 14-day-old mice showed that the dramatic epithelial changes are not present at birth, but become more pronounced with age. At 8 days post partum, the seminiferous epithelium was composed mainly of Sertoli cells (Ser) and spermatogonia (S), whereas the first spermatocytes (Spc) were observed 14 days after birth. In Ehd1$^{R398W/R398W}$ *mice, spermatocytes (Spc) showed more condensed chromatin and an increased number of apoptotic cells (Ap) were seen in the epithelium. Scale bar: 20µm (magnified images) / 500µm (overview images).*

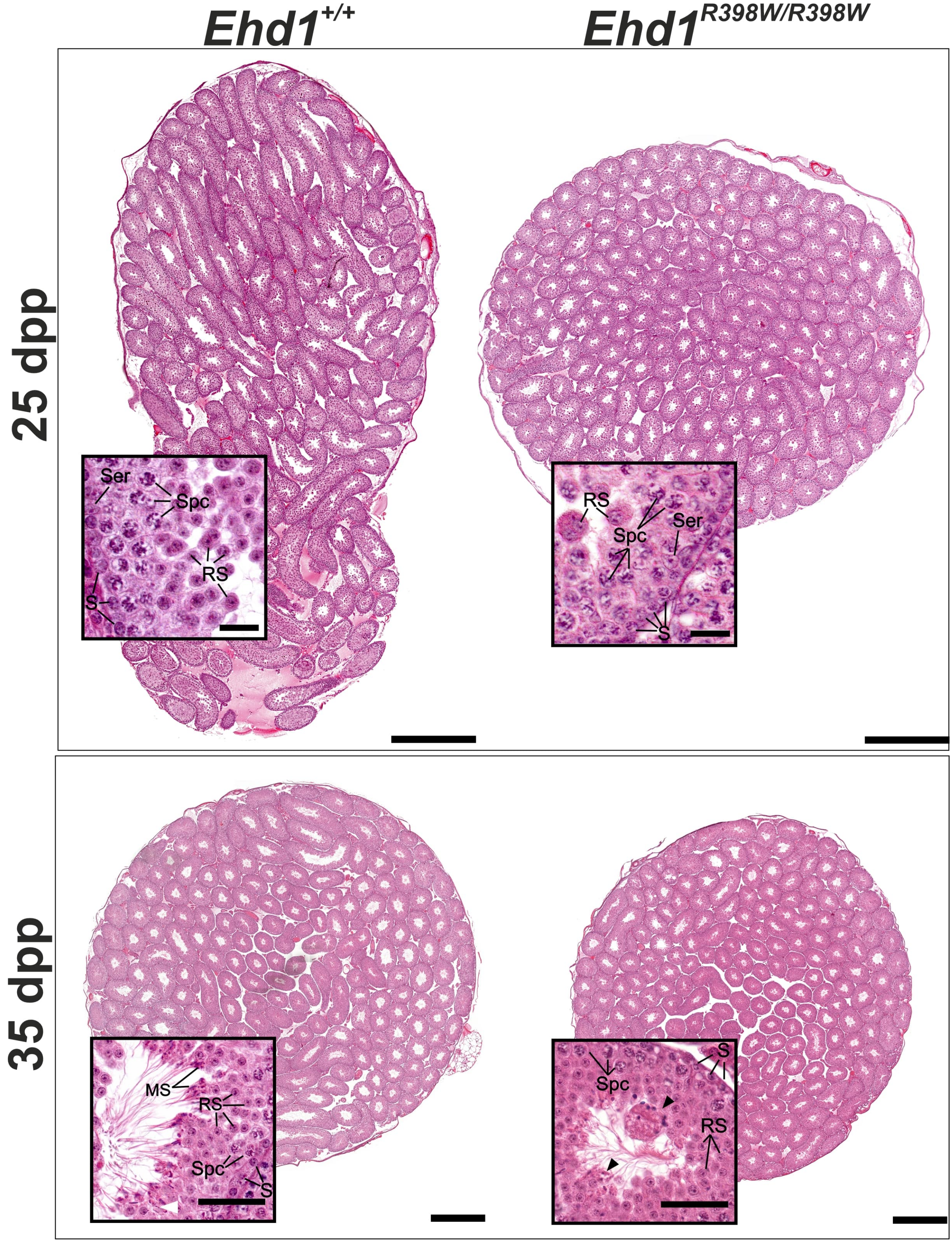

Ehd1+/+
Ehd1R398W/R398W
25 dpp
35 dpp
Ser
Spc
RS
S
MS

SUPPLEMENTARY FIGURE 6. Overview images of testes at 25 and 35 days postpartum

At 25 days of age, the first round spermatids (RS) were visible. By this time, the blood-testis barrier is usually complete and divides the seminiferous epithelium into a basal and an adluminal compartment. Ehd1$^{R398W/R398}$ mice showed a delay in germ cell maturation, with round spermatids observed in fewer tubules compared to wildtype. Additionally, large, round, structures of presumably cytoplasmic origin were observed in the luminal region. These structures were multinucleated, probably containing defective or underdeveloped round spermatids, and could not be observed in the wildtype testis. Within the first 35 days of life, the first spermatogenic wave is completed and the first mature spermatozoa are to be detached from the seminiferous epithelium. Normally, different cell stages, i.e. spermatogonia (S), spermatocytes (Spc), round spermatids (RS), elongating spermatids and mature spermatozoa (MS) have developed and are present in the epithelium. Although the defects observed in young Ehd1$^{R398W/R398W}$ mice were not as severe as in the adults, the first signs of spermatogenic failure were evident as multinucleated structures (arrow head) extended into the tubule lumen, the epithelial architecture appeared disorganized and parts of the sperm tails protruding into the lumen often lacked a connection to condensed nuclei. Scale bar: 20µm (magnified images) / 500µm (overview images).

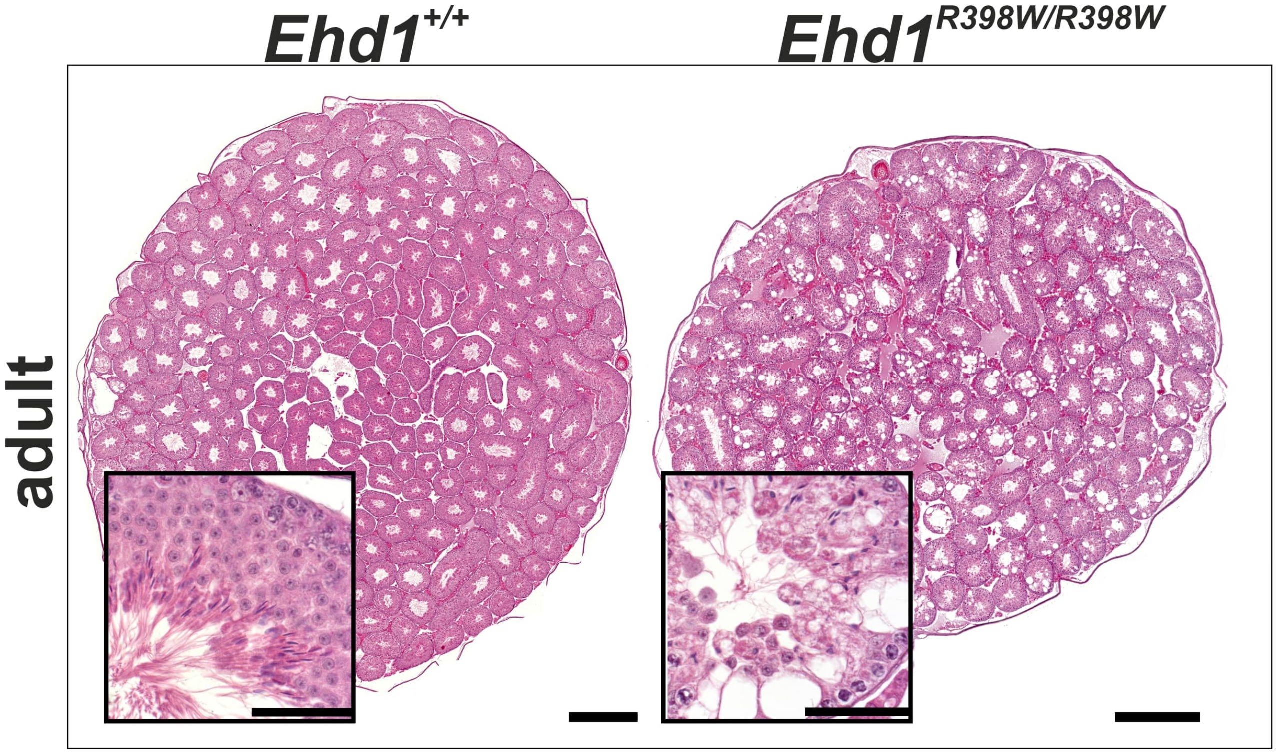

SUPPLEMENTARY FIGURE 7. Overview images of adult testes

Overview HE images of complete testicular cross-sections of adult mice revealed the full extent of destruction, which affected almost all seminiferous tubules in the Ehd1$^{R398W/R398W}$ mouse. Close examination of the magnified areas allowed a clear assignment to the corresponding stages of spermatogenesis for Ehd1$^{+/+}$, as the predominant cell types could be clearly identified. The epithelium appeared homogeneous and parts of the sperm tails were visible in the lumen of the seminiferous tubules. The high degree of epithelial destruction in Ehd1$^{R398W/R398W}$ mice made it impossible to address the spermatogenic stages. The appearance as well as the localisation of certain cell types were altered, suggesting an impairment of the overall spermatogenic process. Scale bar: 50µm (magnified images) / 500µm (overview images).

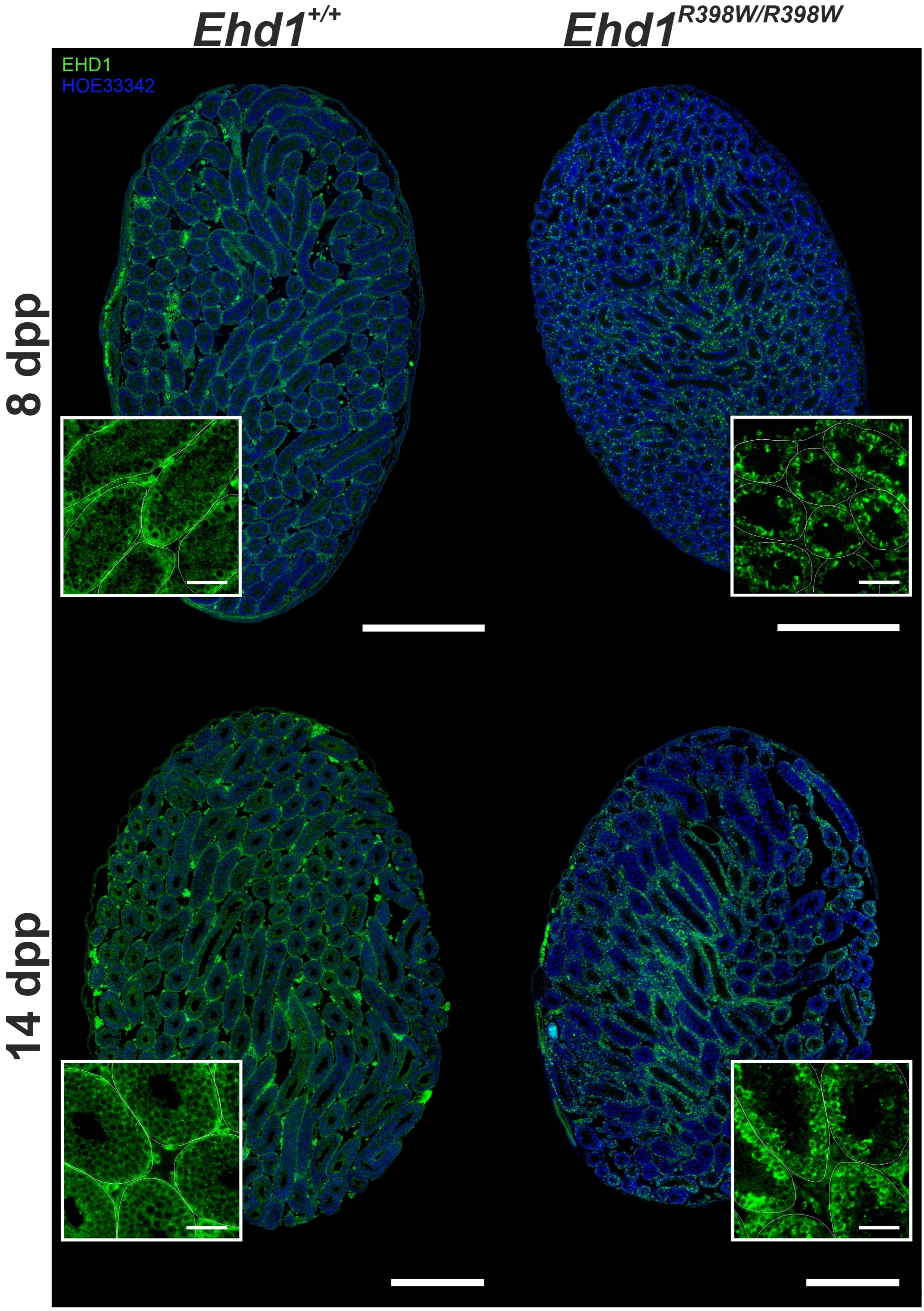

Ehd1+/+
Ehd1R398W/R398W
EHD1
HOE33342
8 dpp
14 dpp

SUPPLEMENTARY FIGURE 8. Overview of EHD1 immunofluorescence at 8 and 14 days postpartum

Immunofluorescence of testicular cross-sections of 8- and 14-days-old Ehd1$^{+/+}$ *and Ehd1*$^{R398W/R398W}$ *mice revealed that EHD1 expression was already altered during the first spermatogenic wave. In 8-day-old mice, wildtype EHD1 appeared homogenously distributed in close proximity to spermatogonial cells near the basement membrane. Although the mutant protein showed the same localization, it formed kind of aggregates. By 14 days of life, the wildtype protein shifted towards the lumen and spread throughout the epithelium, whereas the mutant protein further accumulated in the basal area. Green: EHD1, blue: HOE33342 (nuclei). Scale bar: 50 µm (magnified images), 500 µm (overview images).*

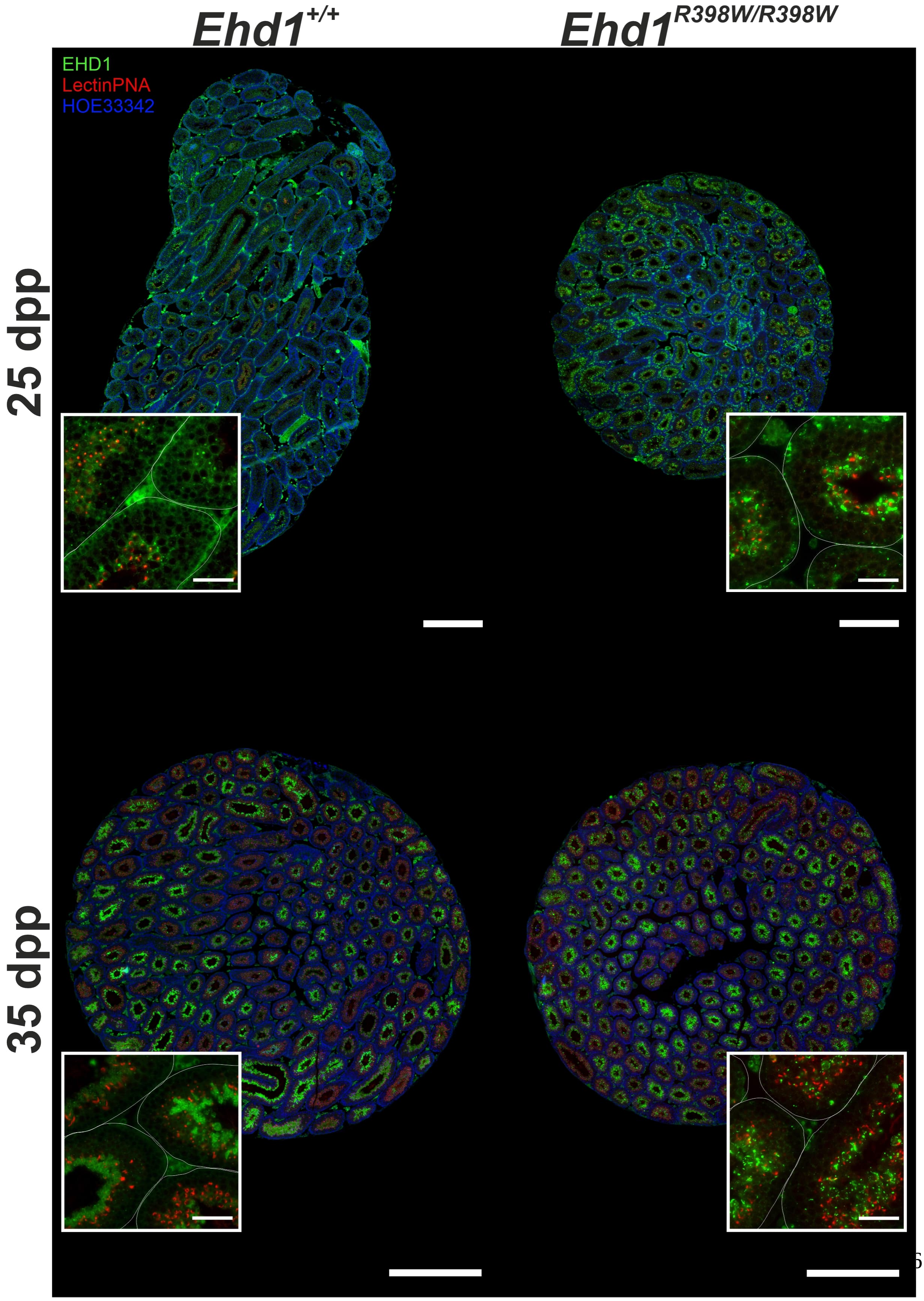
Ehd1+/+
*Ehd1*R398W/R398W
EHD1
LectinPNA
HOE33342
25 dpp
35 dpp

SUPPLEMENTARY FIGURE 9. Overview of EHD1 immunofluorescence at 25 and 35 days postpartum

25 days after birth, the first developing acrosomes were visible. Wildtype EHD1 was observed near the luminal edge as well as in sharply demarcated round structures, some of which interacted with acrosomal structures. In contrast, aggregates of mutant protein did not show any co-localization with the developing acrosomes. 35 days after birth, the first spermatogenic wave is completed. In the Ehd1$^{+/+}$ *mouse, the protein showed a stage-dependent expression pattern, whereas it was difficult to clearly assign the stages of spermatogenesis in the Ehd1*$^{R398W/R398W}$ *mouse. The mutation-induced change in EHD1 function appeared to have a negative effect on early spermatogenic processes, even when the seminiferous epithelium did not yet show severe atrophic defects – these appear to worsen with increasing age. Green: EHD1, red: LectinPNA (acrosomes), blue: HOE33342 (nuclei). Scale bar: 50 µm (zoom images), 500 µm (overview image).*

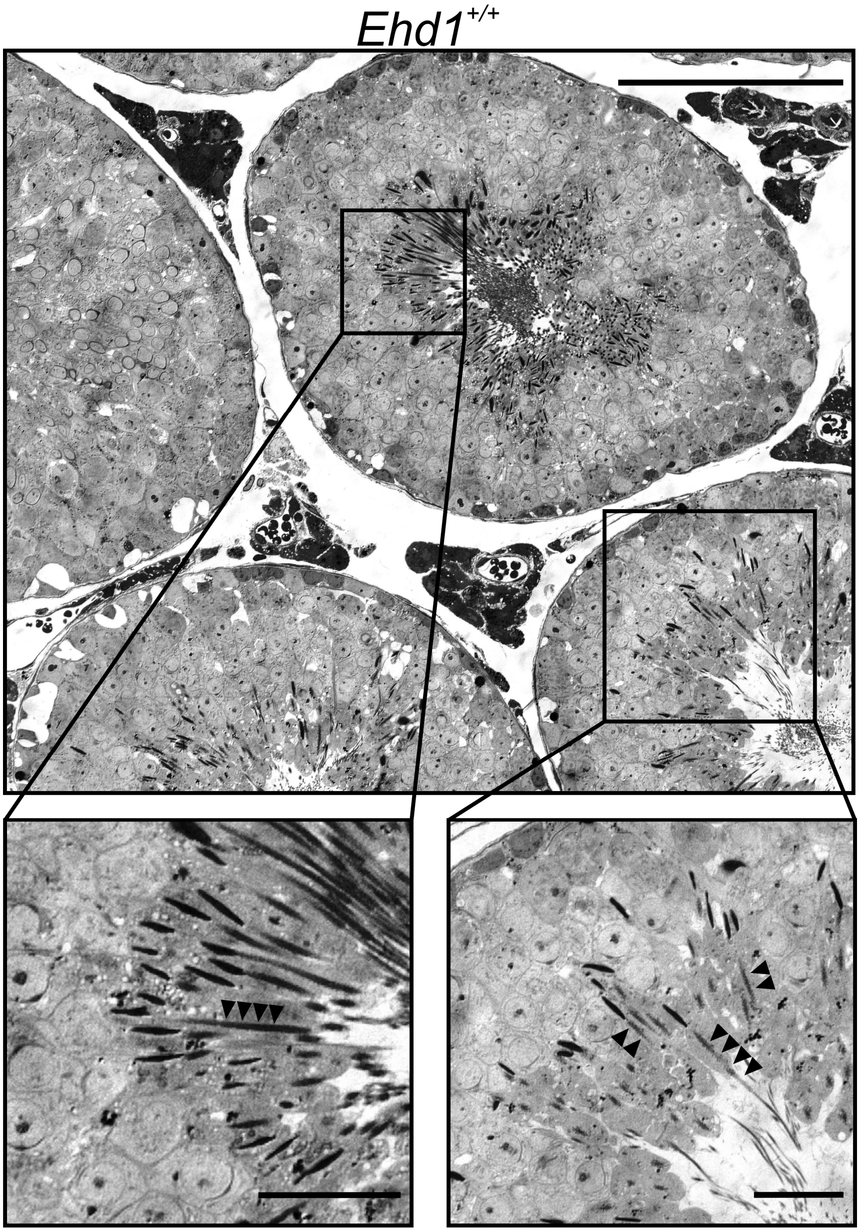

SUPPLEMENTARY FIGURE 10. Semi-thin section of seminiferous tubules of a *EHD1*$^{+/+}$ mouse

The presence of sperm tail midpieces in Ehd1$^{+/+}$ *mice was examined on a semi-thin section of epon-embedded tissue colored with Richardson's methylene blue/Azure II stain (K. C. Richardson, L. Jarett & E. H. Finke (1960), Stain Technology, 35:6, 313-323, DOI:10.3109/10520296009114754). Sperm tail midpieces with the characteristic mitochondrial sheaths (arrowheads) were observed near the luminal edge of the seminiferous tubules‘ cross-sections. Depending on the stage of spermatogenesis, the epithelium exhibits the characteristic developmental stages of the different cell types. Scale bar: 100µm (overview image); 20µm (magnified images).*

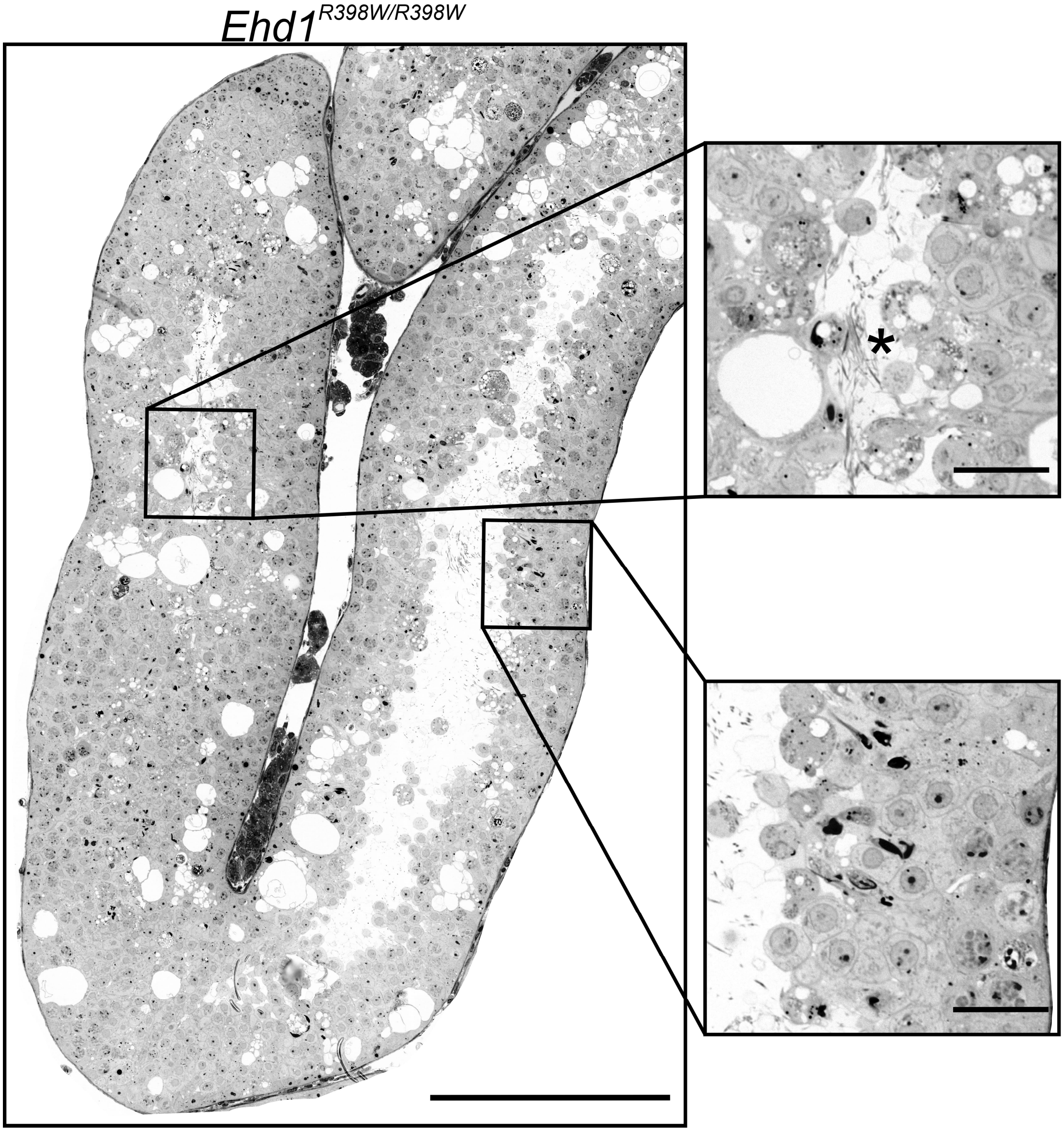

SUPPLEMENTARY FIGURE 11. Semi-thin section of a seminiferous tubule of a *EHD1*$^{R398W/R398W}$ mouse

The presence of sperm tail midpieces in Ehd1$^{R398W/R398W}$ *mice was examined on a semi-thin section of epon-embedded tissue colored with Richardson's methylene blue/Azure II stain (K. C. Richardson, L. Jarett & E. H. Finke (1960), Stain Technology, 35:6, 313-323, DOI:10.3109/10520296009114754). The mutation-induced severe destruction of the epithelium prevented a clear assignment to a specific stage of spermatogenesis in this longitudinal section of a seminiferous tubule. Although sperm tail-like structures (*) were observed in the lumen, these were not associated with elongated spermatid heads and lacked the typical characteristics of sperm tail midpieces. Scale bar: 200µm (overview image); 25 µm (magnified images).*
